# Proton-Coupled Electron Transfer and Hydrogen Tunneling in Olive Oil Phenol Reactions

**DOI:** 10.3390/ijms25126341

**Published:** 2024-06-07

**Authors:** Jelena Torić, Ana Karković Marković, Stipe Mustać, Anamarija Pulitika, Cvijeta Jakobušić Brala, Viktor Pilepić

**Affiliations:** 1 Faculty of Pharmacy and Biochemistry, University of Zagreb, 10000 Zagreb, Croatia; jelenatoric@gmail.com (J.T.); ana.karkovic@pharma.unizg.hr (A.K.M.); stipe.mustac@pharma.unizg.hr (S.M.); 2 Faculty of Chemical Engineering and Technology, University of Zagreb, 10000 Zagreb, Croatia; pulitika@fkit.unizg.hr

**Keywords:** hydroxytyrosol, proton-coupled electron transfer, hydrogen tunneling, kinetic isotope effect, intrinsic bond orbital analysis, average local ionization energy, electron donor Fukui function

## Abstract

Olive oil phenols are recognized as molecules with numerous positive health effects, many of which rely on their antioxidative activity, i.e., the ability to transfer hydrogen to radicals. Proton-coupled electron transfer reactions and hydrogen tunneling are ubiquitous in biological systems. Reactions of olive oil phenols, hydroxytyrosol, tyrosol, oleuropein, oleacein, oleocanthal, homovanillyl alcohol, vanillin, and a few phenolic acids with a DPPH• (2,2-diphenyl-1-picrylhydrazyl) radical in a 1,4-dioxane:water = 95:5 or 99:1 *v*/*v* solvent mixture were studied through an experimental kinetic analysis and computational chemistry calculations. The highest rate constants corresponding to the highest antioxidative activity are obtained for the ortho-diphenols hydroxytyrosol, oleuropein, and oleacein. The experimentally determined kinetic isotope effects (KIEs) for hydroxytyrosol, homovanillyl alcohol, and caffeic acid reactions are 16.0, 15.4, and 16.7, respectively. Based on these KIEs, thermodynamic activation parameters, and an intrinsic bond orbital (IBO) analysis along the IRC path calculations, we propose a proton-coupled electron transfer mechanism. The average local ionization energy and electron donor Fukui function obtained for the phenolic compounds show that the most reactive electron-donating sites are associated with *π* electrons above and below the aromatic ring, in support of the IBO analysis and proposed PCET reaction mechanism. Large KIEs and isotopic values of Arrhenius pre-exponential factor *A*_H_/*A*_D_ determined for the hydroxytyrosol, homovanillyl alcohol, and caffeic acid reactions of 0.6, 1.3, and 0.3, respectively, reveal the involvement of hydrogen tunneling in the process.

## 1. Introduction

Oxidative stress, often induced by an overproduction of free radicals, presents a high risk to human health [1,2]. Free radicals, as reactive compounds, initiate chain reactions, leading to damage to biological molecules such as lipids, proteins, and DNA. Oxidative stress within a lipid environment is particularly important, as lipids react with oxidants faster than proteins and DNA. Primary antioxidants react directly with free radicals, leaving less reactive radicals unable to damage biological molecules. Natural products are an important source of antioxidants, containing a vast diversity of molecules. The study of the relative antioxidative activity of these compounds is important not only to understand their protective effects but also to design efficient strategies against oxidative stress.

Olive oil, as a cornerstone of the Mediterranean diet, and olive oil phenols have attracted considerable attention in recent decades due to their beneficial pharmacological, medicinal, and biochemical properties [3,4,5,6,7]. The molecules that stand out are hydroxytyrosol [8] and its more complex derivatives oleacein [9] and oleocanthal [10,11], which exhibit cardioprotective, neuroprotective, anticancer, immunomodulatory, and other effects, many of which rely on their ability to react as primary antioxidants. The antioxidant activity of olive oil phenols has been extensively studied using various chemical approaches. These include different tests of antioxidative activity (such as DPPH, ORAC, and ABTS assays), accelerated oxidation in a lipid model system (OSI, oxidative stability index), electrochemical methods (flow injection analysis, amperometry, and cyclic voltammetry), calculating the bond dissociation enthalpy and ionization potential of phenolic hydroxy groups, and measuring the effect on LDL oxidation [12,13]. Their positive effects on lipid oxidation are particularly recognized. The European Food Safety Authority has published a health claim regarding the role of olive oil phenols in protecting LDL from oxidation in vivo: “A daily intake of 20 g of olive oil, which contains at least 5 mg of hydroxytyrosol or its derivatives (e.g., oleuropein and tyrosol) provides beneficial effects” [14].

A kinetic approach is very useful for studying trends in antioxidative activity. The rate constant is a measure of reactivity; the faster the reaction, the higher the antioxidant activity. Kinetics also accounts for reaction mechanisms and other important features, such as the tunneling effect [15,16]. Additionally, computational chemistry methods are often used in the study of antioxidative activity [17].

The antioxidative activity of phenol (ArOH) is related to its ability to transfer a hydrogen to a radical (R•) (Equation (1)) much faster than the chain-propagating reaction of peroxidation of organic compounds (X) (Equation (2)) [15,18]:

R• + ArOH → RH + ArO•(1)

R• + X → RH + X•(2)

In reactions with R•, ArOH converts to a phenoxyl radical (ArO•) and gives an unreactive species (RH). The formed phenoxyl radicals are stabilized by resonance, conjugation, and delocalization effects [15,19]. Reactions of phenols with DPPH• (2,2-diphenyl-1-picrylhydrazyl radical) are often used to evaluate the antioxidant activity of phenols [15,18,20,21,22,23]. They serve as a prototype model for the reactions of phenols with peroxyl radicals, ROO•, which are extremely important in biological systems. However, the study of reactions with peroxyl radicals is rather complex because it is necessary to produce a precursor, and reactions are often rapid [24,25]. DPPH• is commercially available, stable, and possesses a strong absorption band in the visible region (*λ*_max_ ≈ 520 nm), making it suitable for an experimental study.

Formal hydrogen atom transfer (electron and proton transfer) from phenol to radical (Equation (1)) may proceed through different mechanisms [15]. Electron transfer (ET) and proton transfer (PT) can occur together in a single chemical step, such as proton-coupled electron transfer (PCET) or hydrogen atom transfer (HAT). In PCET, an electron and a proton are transferred in a single step as two separated particles, while in HAT, an electron and a proton are transferred together as a single entity (a hydrogen atom). The main distinction between these mechanisms is that in HAT, the donor and acceptor sites are the same for both electron and proton, while in PCET, an electron and a proton are transferred from different sets of orbitals. The HAT and PCET mechanisms can be differentiated using computational chemistry methods [26,27]. Another possibility is a consecutive mechanism, such as sequential proton loss electron transfer (SPLET), ET/PT, or PT/ET. Generally, HAT/PCET mechanisms are proposed as predominant in nonpolar solvents, while SPLET is favored in polar solvents due to increased dissociation of phenolic OH groups and greater reactivity of phenolate anions [12,18]. The reactions of phenols with peroxyl radicals (ROO•) usually proceed by a concerted (PCET/HAT) mechanism; thus, reaction rate constants in nonpolar solvents, such as dioxane, are the only useful parameters for predicting the antioxidant ability of ArOH [22]. Although dioxane does not represent a physiological medium taking into account its polarity, it could represent a reaction in physiological conditions because the expected reaction mechanism in dioxane is HAT/PCET [12,18], which is the same mechanism that is proposed for ROO• radicals [22]. However, the reaction of phenols with DPPH•, widely used to evaluate the antioxidative properties of synthetic and natural phenols, is typically performed in methanol, where the proposed mechanism is SPLET. This raises questions about the validity of the conclusions drawn from these studies. 

Hydrogen transfer reactions of phenols have been extensively studied as model reactions, mimicking reactions that involve biological oxidation of tyrosine [28]. Borden et al. used the self-exchange reaction between phenol and phenoxyl radicals as a prototype reaction to demonstrate the difference between PCET and HAT reactions [29]. The PCET mechanism is ubiquitous in biological systems [30,31,32], and it has been proposed for numerous reactions of phenols as well as other antioxidants such as vitamin C and vitamin E, enzyme reactions, the cell respiration process, and complex processes during photosynthesis. PCET is also a reaction mechanism in many chemical and electrochemical processes relevant to solar energy devices [33,34,35]. An interesting recent question is the role of the inverted region in biological pathways [36,37]. 

Another important feature of hydrogen transfer reactions is hydrogen tunneling [38,39,40], which has been observed in PCET reactions of natural antioxidants such as vitamin C and vitamin E [41,42,43]. Nakanishi et al. have recently observed hydrogen tunneling in the reaction between *α*-tocopherol and DPPH• [43].

In this study, we present an experimental and computational kinetic study of the reactions of several olive oil phenols with DPPH• in a nonpolar solvent, 1,4-dioxane. We examined the reactions of olive oil phenols, well-recognized as important bioactive compounds, including the simple phenols hydroxytyrosol [44,45], tyrosol [46], homovanillyl alcohol [47,48], and their more complex derivatives, oleuropein [49], oleacein [9], oleocanthal [50,51], and phenolic acids, including caffeic acid [52], *p*-coumaric acid [53], ferulic acid [54], vanillic acid [55], and vanillin. We have determined the reaction rate constants and proposed the PCET mechanism with the involvement of hydrogen tunneling. To the best of our knowledge, this is the first experimental kinetic study of the antioxidative reactions of the olive oil phenols hydroxytyrosol, tyrosol, oleuropein, oleacein, and oleocanthal. 

## 2. Results and Discussion

**Figure 1:**
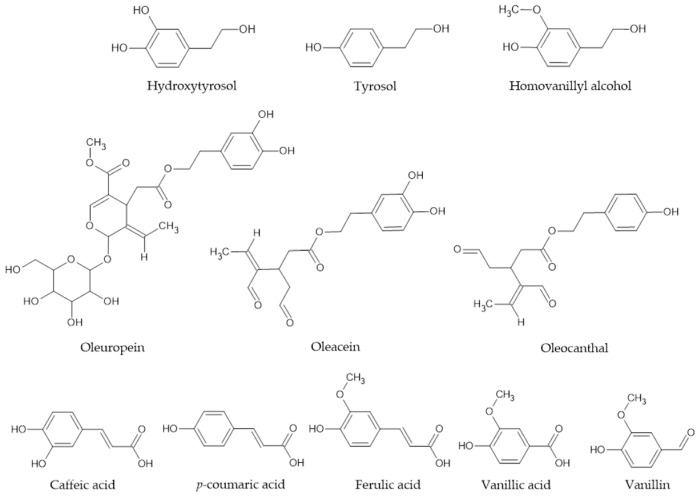
Structures of olive oil phenols.

In this study, we investigated the antioxidant reactions of simple olive oil phenols, hydroxytyrosol (HOTyr), tyrosol, homovanillyl alcohol (HVA), their more complex derivatives, oleuropein, oleacein, oleocanthal, and phenolic acids, caffeic acid, *p*-coumaric acid, ferulic acid, vanillic acid, and vanillin. The structures of these compounds are presented in Figure 1 below. We analyzed reactions with DPPH• (2,2-diphenyl-1picrylhydrazyl radical) in a nonpolar solvent mixture, 1,4-dioxane:water = 99:1 and 95:5 *v*/*v*.

### 2.1. Reaction of Olive Oil Phenols with DPPH•

Rate constants for reactions of phenols with DPPH• were determined by following the disappearance of DPPH• radical at 520 nm (see Supplementary Material, Figures S11 and S12). Kinetic measurements were conducted under pseudo-first-order conditions, with phenol in excess at least ten times. Second-order reaction rate constants, *k*_ArOH_, were obtained from the slopes of plots of the observed pseudo-first-order rate constants vs. the concentration of phenol (see Supplementary Materials, Tables S1–S7, and Figures S1–S7). For each analyzed phenol reaction, the dependence was strongly linear, in accordance with the rate law:

(3) $$-\frac{d\left[DPPH•\right]}{dt}=\frac{1}{2}k_{ArOH}\left[DPPH•\right]\left[ArOH\right]$$

The observed rate constant is as follows:

(4) $$k_{obs}=2k_{ArOH}\left[ArOH\right]$$

The same rate law has been determined previously for reactions of various phenols and flavonoids with DPPH• by others [15]. The overall stoichiometry of the reaction is ArOH:DPPH• = 1:2. The stoichiometry was determined by mixing a small quantity of phenol with an excess of DPPH•. This is related to factor 2 in the rate law. The obtained rate law is consistent with the proposed reaction mechanism [27]. 

In the first slow step (*k*_ArOH_), phenol reduces the DPPH• radical, giving a phenoxyl radical (ArO•) and reduced H-DPPH (Equation (5)):

ArOH + DPPH• → ArO• + H-DPPH (*k*_ArOH_) (5)

The phenoxyl radical formed in the relatively slow first step either reduces another DPPH• or reacts with another molecule of ArO• in the subsequent fast reaction [56]:

ArO• + DPPH• → products (6)

ArO• + ArO• → dimer or disproportionation products (7)

The rate parameters were determined in 1,4-dioxane:water = 99:1 and 95:5 *v*/*v* solvent mixtures. Olive oil phenols are particularly recognized for their antioxidant activity in lipid protection, and such a nonpolar environment can be represented by dioxane as a nonpolar solvent. Solvent impurities can dramatically influence the experimental measurement of the rate parameters of these reactions, as previously observed and explained by the influence of traces of basic and acidic impurities in the solvent [57]. Dioxane appears to be the solvent in which these problems are present to the smallest extent. 

Rate constants and kinetic isotope effects for the reactions of phenols with DPPH• in a 1,4-dioxane:water (99:1 or 95:5 *v*/*v*) solvent mixture are presented in Table 1 and activation parameters in Table 2. 

The highest rate constants are observed for the ortho-diphenols HOTyr, oleuropein, and oleacein. HOTyr is a simple phenol, and oleuropein and oleacein are its more complex derivatives with the same aromatic ring structure (Figure 1). Tyrosol, which lacks one OH group compared to HOTyr, shows a rate constant approximately 300 times smaller than HOTyr. Oleuropein and oleacein, which are complex derivatives of HOTyr, show greater rate constants than oleocanthal, which is a derivative of tyrosol. HVA, with a metoxy group in place of one OH group, has a rate constant ~15 times smaller than that of HOTyr. Similar observations are found in the case of phenolic acids. Caffeic acid, a carboxylic acid with two OH groups in the ortho-position on the aromatic ring, has a rate constant ~200 times larger than that of *p*-coumaric acid, which lacks one OH group compared to caffeic acid. Ferulic acid, with a methoxy group in the ortho-position relative to the OH group, has a rate constant ~3 times smaller than that of caffeic acid. 

The antioxidative activity of the phenols reacting with the hydrogen atom transfer mechanism can be predicted by considering the bond dissociation enthalpy (BDE) of the phenolic OH bond [15]. According to BDE, the most efficient antioxidants are ortho-diphenols, which are related to the stabilization of phenoxy radicals by internal H-bonds [19,58]. The presence of an OH group in the ortho-position decreases BDE by ~40 kJ/mol, while the presence of a methoxy group in the ortho-position slightly stabilizes the radical. The BDE values in the gas phase for HOTyr, tyrosol, and phenol are 307, 343, and 347 kJ/mol, respectively [25]. Tyrosol BDE indicates the stabilizing effect of a –CH_2_CH_2_OH chain in the para-position, and HOTyr BDE reflects the additional effect of the ortho-OH group. BDEs in water and in benzene solutions follow the same trend as in the gas phase [19]. Our experimentally determined rate constants follow the trend in antioxidant activities based on BDE [19].

### 2.2. Reaction Mechanism of Hydrogen Transfer

The reaction mechanism of the reaction between phenol and DPPH• (Equation (5)) involves electron (ET) and proton (PT) transfer. There are a few possible mechanisms for the transfer of electrons and protons. ET and PT could occur concertedly in a single reaction step (the HAT/PCET mechanism) or stepwise in two reaction steps such as PT/ET, ET/PT, or SPLET. The mechanism of ET and PT can be deduced by considering kinetic isotope effects (KIEs) and thermochemical analysis [59].

KIEs in the reactions of phenols with DPPH• were measured in reaction solvent mixtures 1,4-dioxane:H_2_O (*k*_ArOH_) and 1,4-dioxane:D_2_O (*k*_ArOD_) (*v*/*v* = 99:1; 95:5).

KIE = *k*_ArOH_/*k*_ArOD_

ArOD was obtained by dissolving phenol in a 1,4-dioxane-D_2_O solvent mixture due to the fast exchange of phenol OH hydrogens with deuterium. *k*_ArOD_ was determined in the same way as *k*_ArOH_. The kinetic isotope effects are presented in Table 1.

In all the analyzed reactions, we have determined the KIEs. The observed KIEs clearly indicate the involvement of proton transfer in the rate-determining step. In the sequential ET/PT and SPLET mechanisms, the rate-determining step is ET, and so the sequential ET/PT and SPLET mechanisms can be excluded based on the determined KIE. 

Burton et al. determined KIEs in reactions of substituted phenols and peroxyl radicals: 2,3,5,6-tetramethyl-4-methoxyphenol (10.6), 2,6-di-tert-butyl-4-methoxyphenol (9.4), 2,6-di-tert-butyl-4-methylphenol (6.8), and α-tocopherol (4.0) [24]. Small KIEs (2*–*3.3) were also determined in reactions of substituted phenols with DPPH• in cyclohexane [27] and α-tocopherol with DPPH• in water (6.6) [43].

Another possibility is a consecutive mechanism, where the first step is a slow PT, followed by a fast ET. This can be rationalized through thermochemical analysis. In the thermochemical analysis, the standard reaction Gibbs energy for initial PT in the sequential PT/ET is compared to the activation Gibbs energy determined experimentally for the corresponding reaction, with the assumption that the activation Gibbs energy is always at least equal to or greater than the standard reaction Gibbs energy [31]. 

We have determined experimentally activation Gibbs energy and other thermodynamic activation parameters from the temperature dependence of rate constants in a range from 10 to 45 °C (Supplementary Materials, Tables S8–S10 and Figures S8–S10).

The determined activation Gibbs energies for the reactions of HOTyr, HVA, and caffeic acid are 69.0, 82.5, and 74.7 kJ/mol, respectively. A consecutive PT/ET mechanism cannot be excluded as a possible mechanism if the corresponding Δ_r_*G*^ѳ^ is below the value of the activation Gibbs energy. Δ_r_*G*^ѳ^ can be calculated from the p*K*_a_ value of the corresponding phenol as Δ_r_*G*^ѳ^ = −*RT*ln*K*_a_. p*K*_a_ values of the phenolic OH group in water are about 10 or greater. In nonpolar solvents like dioxane, it is reasonable to assume that the p*K*_a_ values are significantly higher than those in water. Rossini et al. studied the influence of nonpolar solvents on the p*K*_a_ value of different phenols and predicted an increase in p*K*_a_ from the value of 10 in water to 18 in methanol and 26 in acetonitrile [60]. It can be expected that p*K*_a_ in 1,4-dioxane is even higher, as it is a more nonpolar solvent than acetonitrile. The highest values of Δ_r_*G*^ѳ^ for HOTyr, HVA, and caffeic acid reactions, which could be expected in the case of PT/ET, are 69.0, 82.5, and 74.7 kJ/mol and correspond to the p*K*_a_ values of 12.1, 14.5, and 13.1, respectively. Considering the previously mentioned assumption for the p*K*_a_ values in 1,4-dioxane, it can be predicted that Δ_r_*G*ʅ is definitely above these values, i.e., Δ*G*^‡^ is lower than Δ_r_*G*^ѳ^. Thus, a sequential PT/ET mechanism can also be excluded. 

The obtained Arrhenius activation parameters are in the same range as those previously reported by Foti et al. for reactions of substituted phenols with DPPH• [27]. 

Altogether, it can be concluded that the mechanism of PT and ET in reactions of analyzed phenols with DPPH• is the concerted PCET or HAT mechanism.

### 2.3. Hydrogen Tunneling

A large KIE, above the value predicted semi-classically, is a typical sign of the involvement of hydrogen tunneling in the reaction [61]. According to Bell, the upper limit for KIE in the case of an OH bond is 8, calculated considering stretching vibrations, and 13 with bending vibrations included. The appearance of a KIE value greater than the upper limit is clear evidence of hydrogen tunneling. In the reactions of HOTyr, HVA, and caffeic acid, we have determined KIEs of 16.0, 15.4, and 16.7, respectively. 

It is interesting that in the case of the structurally similar phenols HOTyr (16.0) and tyrosol (3.3), we have determined significantly different KIEs. In addition, there is a great difference in the rate constants between HOTyr and Tyr, with the HOTyr rate constant being 300-fold greater. This significant difference in reactivity seems to be related to the involvement of hydrogen tunneling in the process [61]. 

KIEs and hydrogen tunneling have been observed earlier in antioxidative reactions, such as the reaction of vitamin C with TEMPO• (KIE~30) [42] or hexacyanoferrate (III) [62].

An additional sign of the involvement of hydrogen tunneling in the process is the deviation in isotopic values of thermodynamic activation parameters *A*_H_/*A*_D_ and Δ*E*_a_ from semi-classically predicted values [61,63]. The semi-classical limits of *A*_H_/*A*_D_ in a hydrogen-transfer process are 0.7–1.2, and the upper limit for Δ*E*_a_ value is 5.1 kJ/mol, calculated from the difference between zero-point energies *E*_0_(D) − *E*_0_(H) for the dissociation of the OH bond. The isotopic values of thermodynamic activation parameters are presented in Table 3. 

The observed *A*_H_/*A*_D_ in the reactions of HOTyr, HVA, and caffeic acid are 0.6, 1.3, and 0.3, respectively, all of which fall outside of the predicted semiclassical limits, supporting the proposed hydrogen tunneling. This is further corroborated by the observed isotopic differences in the activation energy Δ*E*_a_, 8.1, 5.9, and 9.5 for HOTyr, HVA, and caffeic acid, respectively, also beyond the semiclassical limit of 5.1 kJ/mol. Hydrogen tunneling appears to be an important feature of the reactions of phenols with prominent antioxidative activity.

### 2.4. Computational Analysis

**Figure 2:**
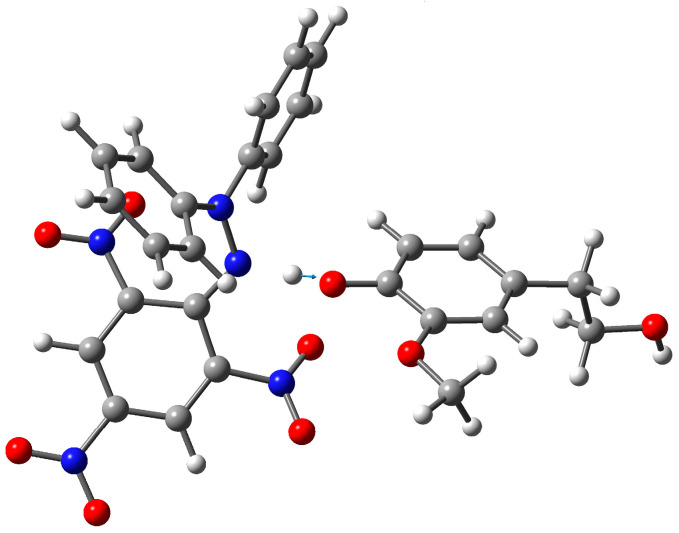
Transition structure for the proposed hydrogen transfer from HVA to DPPH• obtained from the DFT calculations. The normal mode displacement vector shown (blue arrow) for the unique imaginary frequency of 1608.7*i* cm^−1^ is associated with a motion of the H atom of the O-H moiety of HVA to the N atom of DPPH•.

**Figure 3:**
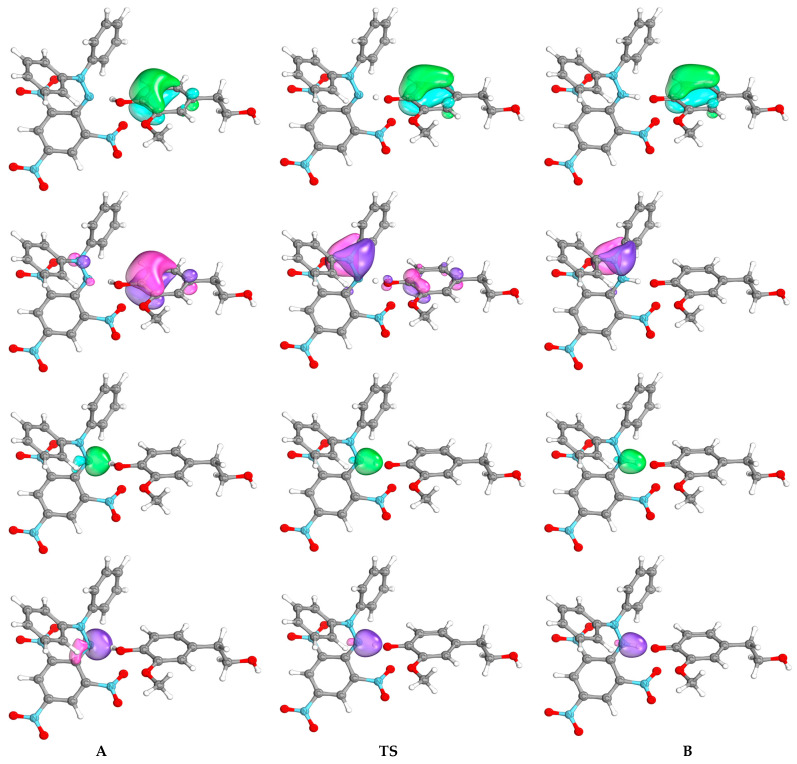

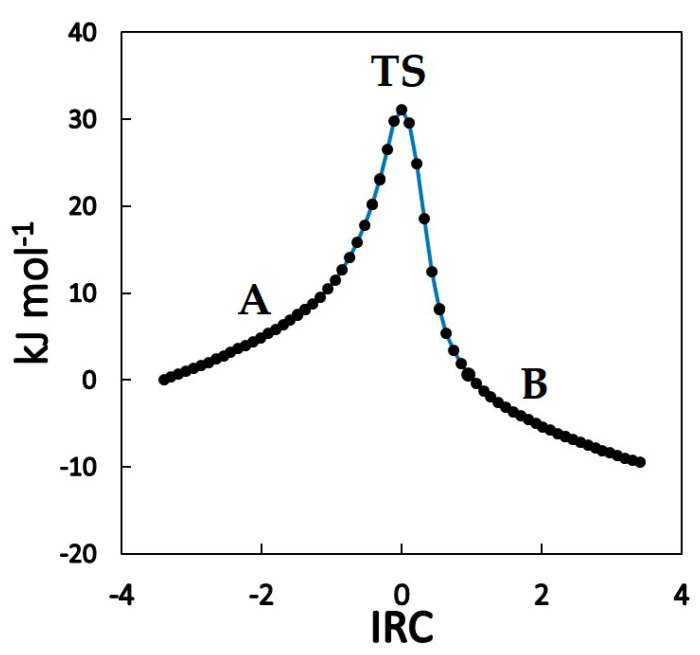
Changes in energy plotted along the IRC path (inset), α (green), and β (purple) intrinsic bond orbitals (IBO) involved in hydrogen transfer from HVA to DPPH• plotted along the intrinsic reaction coordinate (IRC) obtained from the DFT calculations. For IBO analysis along the entire IRC, see also the corresponding video files in the Supplementary Materials.

**Figure 4:**
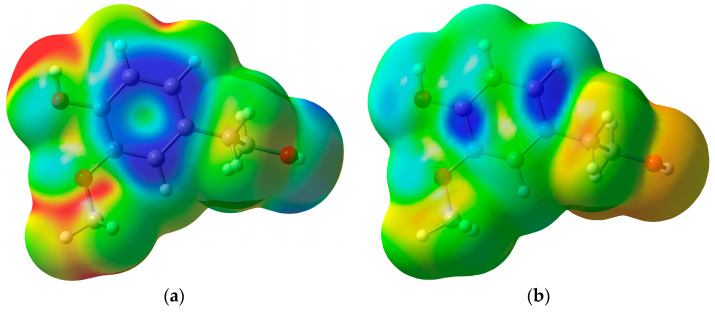
The average local ionization energy *Ī*(*r*) (**a**) and the electron donor Fukui function *f* ¯(*r*) (**b**) plotted on 0.001 a.u. electron density isosurface of HVA. The color scales correspond to the range of values 13.6 eV (red) to 4.5 eV (blue) for *Ī*(*r*) and 0.0 a.u. (red) to 0.0003 a.u. (blue) for *f* ¯(*r*). The lowest values of *Ī*(*r*) (blue areas) and the highest values of *f* ¯(*r*) (blue areas) indicate the most preferred sites for donating electrons. The lowest average local ionization energy *Ī*_S,min_ has a value of 8.64 eV.

The calculations of transition state structures (TS) for the proposed hydrogen transfer from HOTyr, HVA, and caffeic acid to DPPH• were performed and further explored with an intrinsic reaction coordinate (IRC) [64] and intrinsic bond orbital (IBO) analysis [65,66,67]. The obtained results are summarized in Figure 2, Figure 3 and Figure 4 and Table 4 and Table 5, and also in the Supplementary Materials, Figures S13–S17 and Table S11. Compared to related TS structures for hydrogen transfer from phenol or *p*-metoxyphenol to DPPH• obtained by Foti et al. [27], the obtained TS structures for HVA (Figure 2), HOTyr, and caffeic acid (Figure S13) have slightly shorter H⋯O and a bit longer N⋯H distances (Table 4). The calculated Gibbs activation energy (Table 5) agrees very well for HVA with those obtained from the experiment (Table 2) in support of the proposed TS structure. The reasonable agreement between the calculated and experimental Gibbs activation energies for HOTyr and caffeic acid can be explained by the presence of a second OH group. The calculated KIEs, when corrected for tunneling, are close to the experimentally obtained values (Table 5).

Intrinsic bond orbital (IBO) analysis proposed by Knizia [66] enables the visualization of molecular orbitals and electronic structure changes in an intuitive way along reaction paths [65,66,67]. The IBO analysis proved to be successful in the interpretation of reaction mechanisms and also in reactions of hydrogen transfer, where it was shown that it can unambiguously distinguish between HAT and PCET processes [67,69,70]. The representative intrinsic bond orbitals (IBOs) involved in hydrogen transfer from HVA, HOTyr, and caffeic acid to DPPH• plotted along the intrinsic reaction coordinate (IRC) obtained from the DFT calculations are shown in Figure 3, Figures S14 and S15, respectively. For IBO analysis along the entire IRC, see also the corresponding video files in the Supplementary Materials. In all three cases, the electron transferred to the DPPH• N atom comes from the aromatic ring orbital of the phenolic compound, leaving an unpaired electron on it, while the proton from the phenolic OH group is transferred separately to the orbitals located on another N atom of DPPH•. It can be concluded that in these cases, proton transfer (PT) and electron transfer (ET) are separate from different reaction sites and involve different orbitals, which clearly points to PCET reactions.

Average local ionization energy *Ī*(*r*) [71] and electron donor Fukui function *f*¯(*r*) [72] are local reactivity descriptors used to identify molecular reactivity sites for reactions with electrophiles or radicals in a series of studies [73,74,75,76,77,78]. The *Ī*(*r*) and *f*¯(*r*) obtained for HVA are shown in Figure 4 and for HOTyr and caffeic acid in Figures S16 and S17. For all of these phenolic compounds, the most reactive electron-donating sites (blue areas) are associated with π electrons above and below the aromatic rings, corresponding to the phenol orbital from which the electron is transferred to DPPH•, as obtained in the IBO analysis.

## 3. Materials and Methods

1,4-dioxane, 2,2-diphenyl-1-picrylhydrazyl (DPPH•), homovanillyl alcohol, tyrosol, caffeic acid, *p*-coumaric acid, HCl (1 M, Titrisol), and DCl (99% D) were obtained from Merck, KGaA, (Darmstadt, Germany), and vanillic acid, ferulic acid, and vanillin were from Fluka Chemie GmbH (Buchs, Switzerland). Hydroxytyrosol and oleuropein were purchased from Extrasynthese (Genay, France), and oleacein and oleocanthal from PhytoLab GmbH & Co. KG (Vestenbergsgreuth, Germany). All chemicals used were of analytical grade. Water and heavy water (Aldrich, 99.9%) were twice distilled and carbon dioxide- and oxygen-free (bubbled with 99.999% N_2_). 

Kinetic Measurements. Pseudo-first-order rate constants for the reaction of phenols with the DPPH• radical have been determined spectrophotometrically by monitoring the decrease in absorbance of DPPH• at 525 nm. An Avantes AvaSpec 2048L StarLine spectrometer (Avantes B.V., Apeldoorn, The Netherlands) provided with a Quantum Northwest QPOD Temperature-Controlled Sample Compartment for Fiber Optic Spectroscopy was used throughout to collect spectral and absorbance-time data. Kinetic measurements were performed under carefully maintained temperature conditions (within the limits of ±0.1 °C). In a typical kinetic run, at least 200 pairs of absorbance-time data were collected and fitted to the common least-squares algorithm. At least three to four observed pseudo-first rate constants were always used to calculate the corresponding rate parameters under the specified reaction conditions. The measurements were performed under pseudo-first-order conditions, taking phenol concentrations to be at least 15-fold in excess, except in the case of secoiridoids, which were 2.5- to 12-fold in excess at their lowest concentrations. Very good pseudo-first-order kinetics were obtained throughout. The stock solutions of reactants were usually prepared by dissolving phenol or DPPH• in 1,4-dioxane. The reaction solution was thermostated for 20 min before the kinetics were started. The reaction was initiated by the addition of an appropriate volume of DPPH• stock solution to a thermostated reaction solution. Measured kinetic isotope effects are corrected considering the content of H in the 1,4-dioxane-D_2_O solvent mixture.

Computational method details. The DFT calculations were carried out using the GAUSSIAN 16 software package [79] at the (U)B3LYP/6-311++G(2d,2p) level of theory, applying very tight convergence criteria. Non-specific solvent effects have been estimated by using the polarizable continuum model (PCM) of the self-consistent reaction field (SCRF) method [80] with 1,4-dioxane as a solvent. The stationary points obtained by geometry optimizations were confirmed either as minima or saddle points by vibrational analysis at the same theory level. The obtained frequencies were scaled by a factor of 0.9679 [68]. The transition state structures were further explored with an intrinsic reaction coordinate (IRC) analysis [64] at the same theory level. The obtained reaction path structures were examined with an intrinsic bond orbital (IBO) analysis utilizing PBE/def2-TZVPPD and the IBO localization procedure [65,66,67] with the IBOVIEW program [81]. Local reactivity descriptors, the average local ionization energy, and the electron donor Fukui function have been calculated from the wave functions in the checkpoint files obtained from the above B3LYP/6-311++G(2d,2p) calculations by the MULTIWFN program [82]. An estimation of the tunneling correction for the calculated reaction constants and KIE was performed from a vibration analysis using the Wigner method [83] with the TAMkin program [84].

## 4. Conclusions

Olive oil *ortho-*diphenols, hydroxytyrosol, oleuropein, and oleacein show the highest antioxidant activity, with the highest values of reaction rate constants. The electron and proton transfers in these reactions proceed via a proton-coupled electron transfer mechanism. Additionally, large kinetic isotope effects have been observed in the reactions of hydroxytyrosol, homovanillyl alcohol, and caffeic acid, indicating the involvement of hydrogen tunneling in the process. The intrinsic bond orbital (IBO) analysis along the IRC path calculations supports the proposed reaction mechanism. The average local ionization energy and electron donor Fukui function obtained for the phenolic compounds show that the most reactive electron-donating sites are associated with *π* electrons above and below the aromatic ring, in support of the IBO analysis and the proposed PCET reaction mechanism. 

## Figures and Tables

**Table 1:** Rate constants (*k*_ArOH_) and kinetic isotope effects (KIEs) for the reaction of phenol and DPPH• in 1,4-dioxane:water solvent mixtures (0.99:0.01 *v*/*v* or 0.95:0.05 *v*/*v*) at 25 °C.

Phenol	*k*_ArOH_/M^−1^ s^−1^	KIE
Simple phenols		
Hydroxytyrosol	2.56 (0.07)	16.0 (1.0)
Tyrosol	0.0083 (0.0007)	3.3 (0.4)
Homovanillyl alcohol	0.150 (0.002)	15.4 (0.3)
Secoiridoids		
Oleuropein	3.03 (0.10)	3.2 (0.4)
Oleacein	2.83 (0.36)	1.7 (0.8)
Oleocanthal	0.88 (0.05)	1.8 (0.2)
Phenolic acids		
Caffeic acid	0.537 (0.009)	16.7 (0.5)
*p*-coumaric acid ^1^	0.0025 (0.0001)	3.4 (0.02)
Ferulic acid ^2^	0.161 (0.029)	
Vanillic acid ^2^	0.0058 (0.0001)	4.1 (0.1)
Vanillin	0.00291 (0.00001)	

^1^ 0.2 M *p*-coumaric acid; ^2^ 0.1 M phenol.

**Table 2:** Thermodynamic activation parameters for the reaction of phenol and DPPH• in 1,4-dioxane-H_2_O solvent mixtures (0.99:0.01 *v*/*v* or 0.95:0.05 (*v*/*v*)) at 25 °C.

Phenol	Δ*G*^‡^/kJ mol^−1^	Δ*H*^‡^/kJ mol^−1^	Δ*S*^‡^/J K^−1^ mol^−1^	*E*_a_/kJ mol^−1^	ln(*A*/M^−1^ s^−1^)
HOTyr	69.0 (0.8)	40.0 (0.7)	−146.0 (2.2)	45.2 (0.3)	16.0 (0.1)
HVA	82.5 (0.9)	33.1 (1.2)	−143.8 (4.2)	35.6 (1.2)	13.1 (0.5)
caffeic acid	74.7 (1.2)	40.8 (0.9)	−113.4 (2.9)	43.4 (0.9)	16.8 (0.3)

**Table 3:** Isotopic difference in activation parameters for the reaction of phenol and DPPH• in 1,4-dioxane-H_2_O solvent mixtures (0.99:0.01 *v*/*v* or 0.95:0.05 *v*/*v*) at 25 °C.

Phenol	ΔΔ*G*^‡^/kJ mol^−1^	Δ*E*_a_ (D, H)/kJ mol^−1^	*A*_H_/*A*_D_
HOTyr	6.9 (3.1)	8.1 (3.0)	0.6
HVA	6.5 (1.8)	5.9 (1.4)	1.3
caffeic acid	6.8 (1.8)	9.5 (1.3)	0.3

**Table 4:** Selected properties of the transition state structures obtained for hydrogen transfer from the 4-OH moiety of the phenols to the DPPH• N atom obtained from the DFT calculations.

Phenol	N⋯H/Å ^a^	H⋯O/Å ^b^	N⋯H⋯O/° ^c^	*v*_H_/cm^−1 d^	*v*_D_/cm^−1 d^
HOTyr	1.302	1.177	163.3	1646.6*i*	1218.8*i*
HVA	1.313	1.167	162.5	1608.7*i*	1192.4*i*
caffeic acid	1.275	1.202	162.5	1689.0*i*	1246.9*i*

^a^ Distance between the N atom of DPPH• and the H atom. ^b^ Distance between the O atom of the phenol and the H atom. ^c^ N⋯H⋯O angle. ^d^ Normal mode imaginary frequency associated with the proton or deuterium transfer scaled by a factor of 0.9679 [68].

**Table 5:** Activation and reaction Gibbs energy and KIE for hydrogen transfer from the 4-OH moiety of the phenols to the DPPH• N atom obtained from the DFT calculations.

Phenol	Δ*G*^‡^_H_/kJ mol^−1^	Δ*G*^‡^_D_/kJ mol^−1^	Δ_r_*G*/kJ mol^−1^	KIE_SC_^a^	KIE_TUNN_^b^
HOTyr	+79.0	+83.4	−1.3	6.0	18.9
HVA	+81.5	+85.6	10.3	5.2	19.2
caffeic acid	+77.5	+83.4	−2.6	4.9	19.6

^a^ Kinetic isotope effects obtained from the calculated Δ*G*^‡^ for H and D substituted reactants. ^b^ Kinetic isotope effects corrected for H atom tunneling using the Wigner method.

## Data Availability

The original contributions presented in the study are included in the article/Supplementary Materials, further inquiries can be directed to the corresponding author/s.

## Supplementary Materials

The supporting information can be downloaded at https://www.mdpi.com/article/10.3390/ijms25126341/s1.
