# Supplementary material for: Proton-Coupled Electron Transfer and Hydrogen Tunneling in Olive Oil Phenol Reactions
Source: Int J Mol Sci. 2024 Jun 7;25(12):6341. doi: 10.3390/ijms25126341 (PMC11203655; doi:10.3390/ijms25126341)
Supplement: Supplementary file 1 [file ijms-25-06341-s001.zip › ijms-3012168_R1_Supplementary_Materials.pdf]

Reaction Rate Constants

Reaction of Hydroxytyrosol and DPPH• Radical

Table S1. Pseudo-first Order Constants (k_{obs}) for Reaction of Hydroxytyrosol and DPPH• Radical in 1,4-Dioxane-H_2O Solvent Mixture (0.99 : 0.01 *v*/*v*) at 25°C.

c(HOTyr) / mol dm^{-3}	$k_{obs} \cdot 10^3$ / s^{-1}
0.001	4.05 (0.44)
0.0013	6.35 (0.43)
0.0015	7.81 (0.36)
0.0020	10.16 (0.60)
0.0028	14.49 (0.43)

c(DPPH•) = 0.00006 mol dm^{-3}.

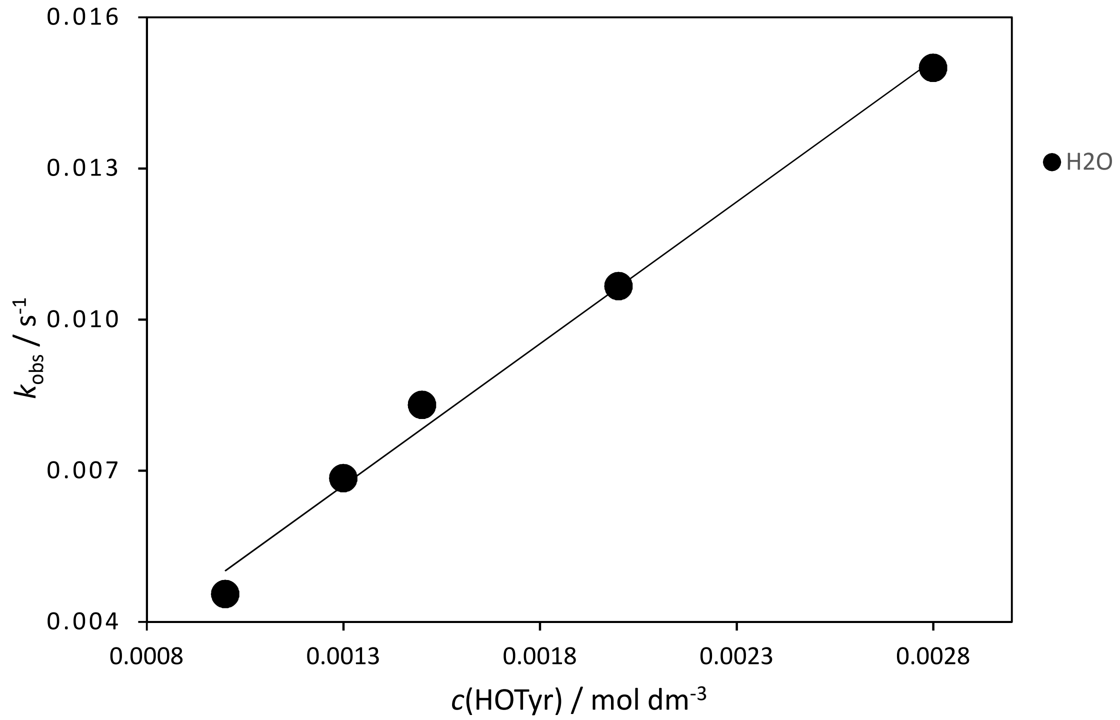

Figure S1. The plot of pseudo-first order constants (k_{obs}) *vs* concentration of Hydroxytyrosol for the reaction of Hydroxytyrosol and DPPH• radical in 1,4-dioxane-H_2O solvent mixture (0.99 : 0.01 *v*/*v*) at 25 °C (R^2=0.9923); *c*(DPPH•) = 0.00006 mol dm^{-3}. Data from Table S1.

Reaction of Tyrosol and DPPH• Radical

Table S2. Pseudo-first Order Constants (k_{obs}) for Reaction of Tyrosol and DPPH• Radical in 1,4-Dioxane-H_2O Solvent Mixture (0.95 : 0.05 *v*/*v*) at 25°C.

c(tyrosol) / mol dm^{-3}	k_{obs}·10^3 / s^{-1}
0.1	0.50 (0.04)
0.2	1.55 (0.11)
0.3	3.82 (0.15)

c(DPPH•) = 0.00006 mol dm^{-3}

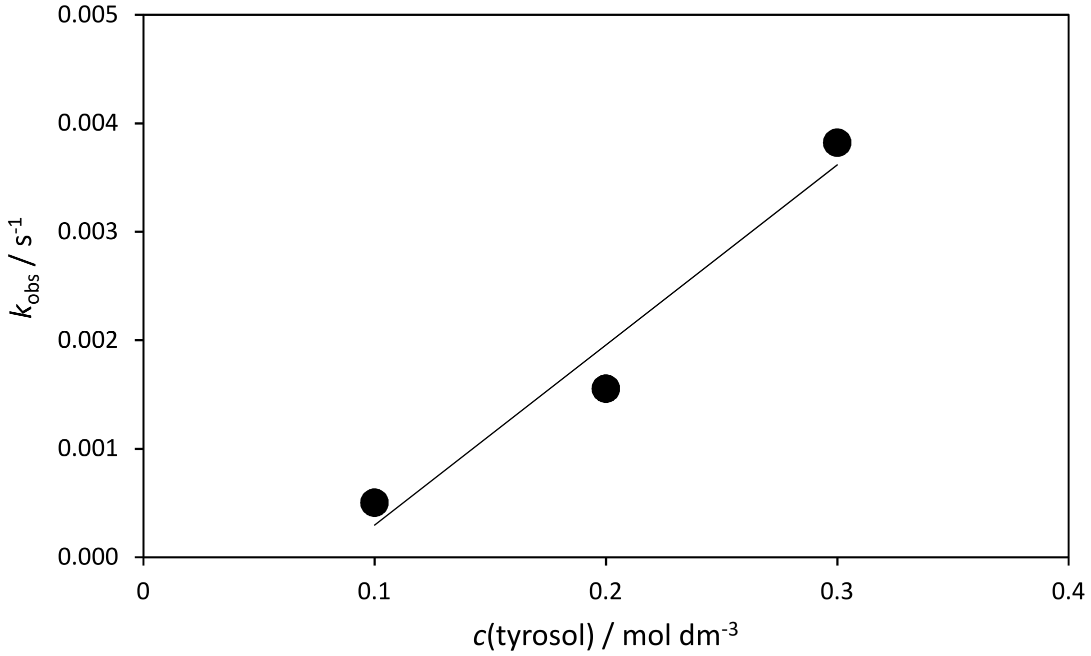

Figure S2. The plot of pseudo-first order constants (k_{obs}) vs. concentration of tyrosol for the reaction of tyrosol and DPPH• radical in 1,4-dioxane-water solvent mixture (0.95 : 0.05 *v*/*v*) at 25 °C (R^2=0.9569); *c*(DPPH•) = 0.00006 mol dm^{-3}. Data from Table S2.

Reaction of Homovanillyl Alcohol and DPPH• Radical

Table S3. Pseudo-first Order Constants (k_{obs}) for Reaction of Homovanillyl Alcohol and DPPH• Radical in 1,4-Dioxane-H_2O Solvent Mixture (0.99 : 0.01 *v*/*v*) at 25°C.

c(HVA) / mol dm^{-3}	$k_{obs}\cdot 10^3$ / s^{-1}
0.005	1.510 (0.018)
0.01	3.300 (0.023)
0.025	7.549 (0.100)
0.05	15.10 (0.30)
0.10	31.40 (0.50)

c(DPPH•) = 0.00006 mol dm^{-3}

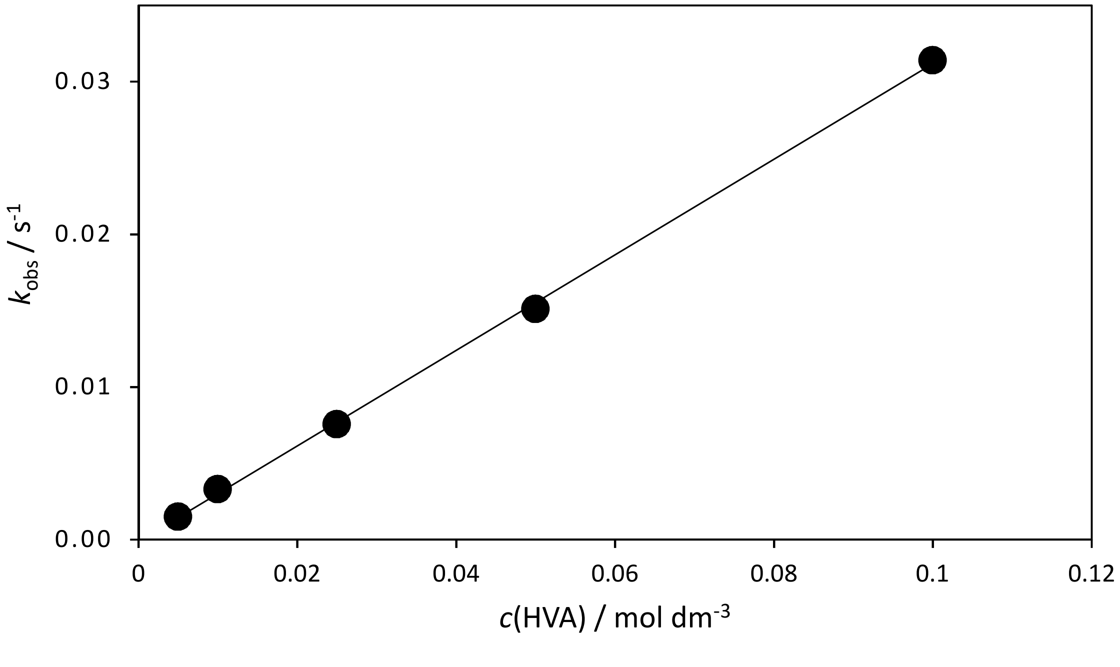

Figure S3. The plot of pseudo-first order constants (k_{obs}) vs. concentration of homovanillyl alcohol for the reaction of homovanillyl alcohol and DPPH• radical in 1,4-dioxane-water solvent mixture (0.99 : 0.01 *v*/*v*) at 25 °C (R^2=0.9994); c(DPPH•) = 0.00006 mol dm^{-3}. Data from Table S3.

Reaction of Oleuropein and DPPH• Radical

Table S4. Pseudo-first Order Constants (k_{obs}) for Reaction of Oleuropein and DPPH• Radical in 1,4-Dioxane-Water Solvent Mixture (0.95 : 0.05 *v*/*v*) at 25°C.

c(oleuropein) / mol dm^{-3}	**H_2O** $k_{obs} \cdot 10^3$ / s^{-1}	**D_2O** $k_{obs} \cdot 10^3$ / s^{-1}
0.0005	1.66 (0.37)	0.95 (0.46)
0.0010	4.31 (0.39)	1.53 (0.93)
0.0017	8.63 (0.49)	3.53 (1.21)
0.0030	16.7 (1.07)	5.58 (1.22)

c(DPPH•) = 0.00006 mol dm^{-3}

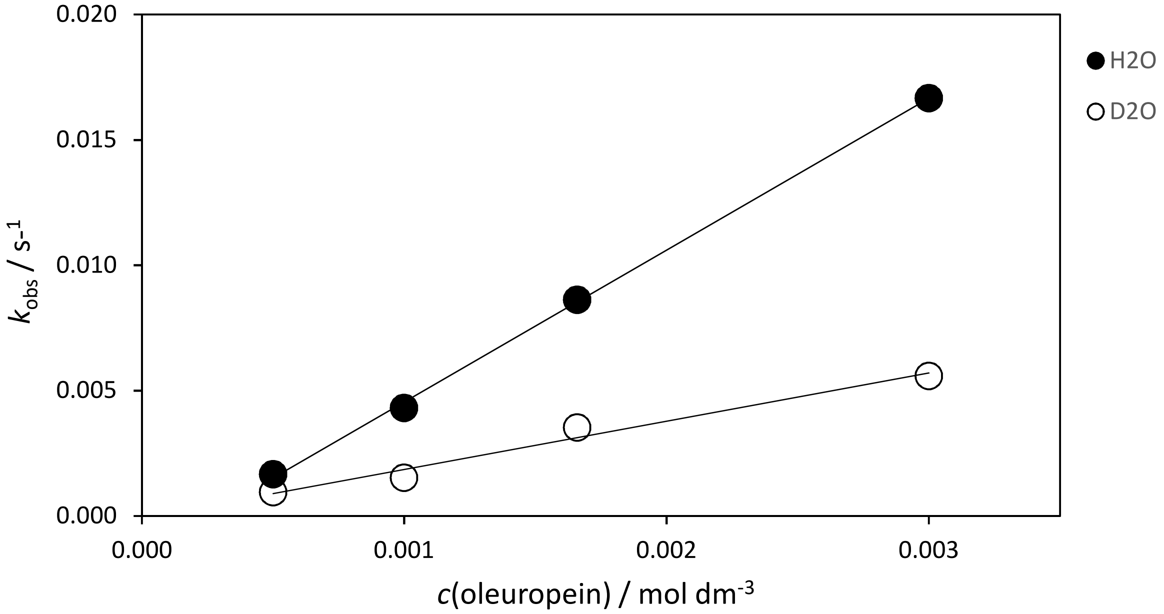

Figure S4. The plot of pseudo-first order constants (k_{obs}) vs. concentration of oleuropein for the reaction of oleuropein and DPPH• radical in 1,4-dioxane-water solvent mixture (0.95 : 0.05 *v*/*v*) at 25 °C (H: R^2=0.9994; D: R^2=0.9786); *c*(DPPH•) = 0.00006 mol dm^{-3}. Data from Table S4.

Reaction of Oleacein and DPPH• Radical

Table S5. Pseudo-first Order Constants (k_{obs}) for Reaction of Oleacein and DPPH• Radical in 1,4-Dioxane-Water Solvent Mixture (0.95 : 0.05 *v*/*v*) at 25°C.

c(oleacein) / mol dm^{-3}	H_2O<br>$k_{obs} \cdot 10^3$ / s^{-1}	D_2O<br>$k_{obs} \cdot 10^3$ / s^{-1}
0.00015	0.91 (0.56)	/
0.0003	1.51 (0.34)	0.82 (0.42)
0.0006	4.05 (0.29)	1.69 (0.97)
0.001	5.51 (1.69)	3.21 (1.93)

c(DPPH•) = 0.00006 mol dm^{-3}

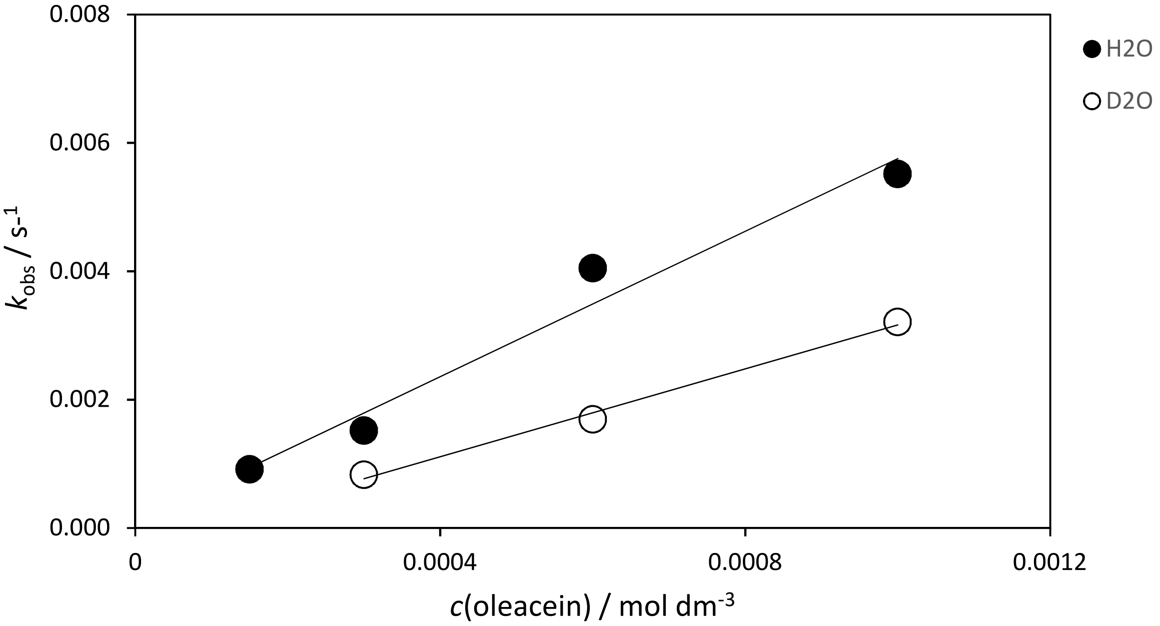

Figure S5. The plot of pseudo-first order constants (k_{obs}) *vs*. concentration of oleacein for the reaction of oleacein and DPPH• radical in 1,4-dioxane-water solvent mixture (0.95 : 0.05 *v*/*v*) at 25 °C (H: R^2=0.9681; D: R^2=0.9944); c(DPPH•) = 0.00006 mol dm^{-3}. Data from Table S5.

Reaction of Oleocanthal and DPPH Radical

Table S6. Pseudo-first Order Constants (k_{obs}) for Reaction of Oleocanthal and DPPH• Radical in 1,4-Dioxane-Water Solvent Mixture (0.95 : 0.05 *v*/*v*) at 25°C.

c(oleocanthal) / mol dm^{-3}	**H_2O** $k_{obs} \cdot 10^3$ / s^{-1}	**D_2O** $k_{obs} \cdot 10^3$ / s^{-1}
0.0005	0.95 (0.03)	0.79 (0.00)
0.00075	1.42 (0.05)	/
0.001	1.91 (0.16)	1.19 (0.09)
0.0015	2.71 (0.12)	1.75 (0.09)

c(DPPH•) = 0.00006 mol dm^{-3}

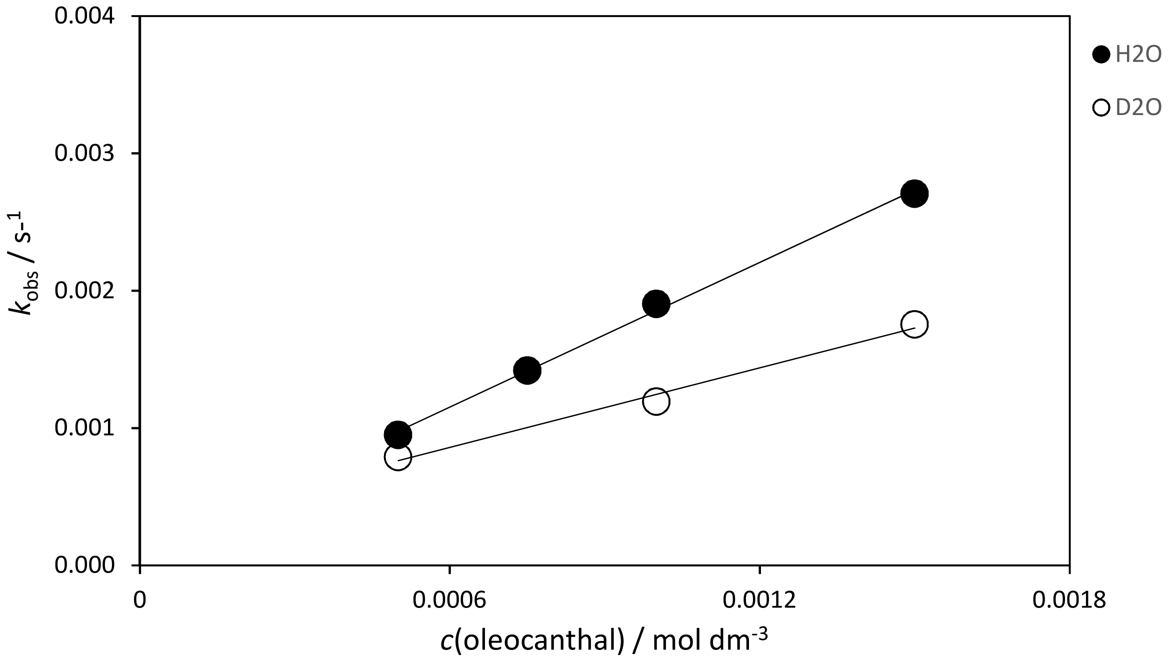

Figure S6. The plot of pseudo-first order constants (k_{obs}) *vs.* concentration of oleocanthal for the reaction of oleocanthal and DPPH• radical in 1,4-dioxane-water solvent mixture (0.95 : 0.05 *v*/*v*) at 25 °C (H: R^2=0.9977; D: R^2=0.9908); *c*(DPPH•) = 0.00006 mol dm^{-3}. Data from Table S6.

Reaction of Caffeic Acid and DPPH• Radical

Table S7. Pseudo-first Order Constants (k_{obs}) for Reaction of Caffeic Acid and DPPH• Radical in 1,4-Dioxane-Water Solvent Mixture (0.95 : 0.05 *v*/*v*) at 25°C.

c(caffeic acid) / mol dm^{-3}	**H_2O** $k_{obs} \cdot 10^3$ / s^{-1}	**D_2O** $k_{obs} \cdot 10^4$ / s^{-1}
0.0012	1.32 (0.07)	0.77 (0.03)
0.0025	2.73 (0.12)	1.61 (0.13)
0.005	5.37 (0.09)	3.22 (0.07)
0.0075	8.12 (0.08)	5.01 (0.13)
0.01	10.7 (0.37)	6.76 (0.22)

c(DPPH•) = 0.00008 mol dm^{-3}; *c*(HCl or DCl) = 0.0005 mol dm^{-3}

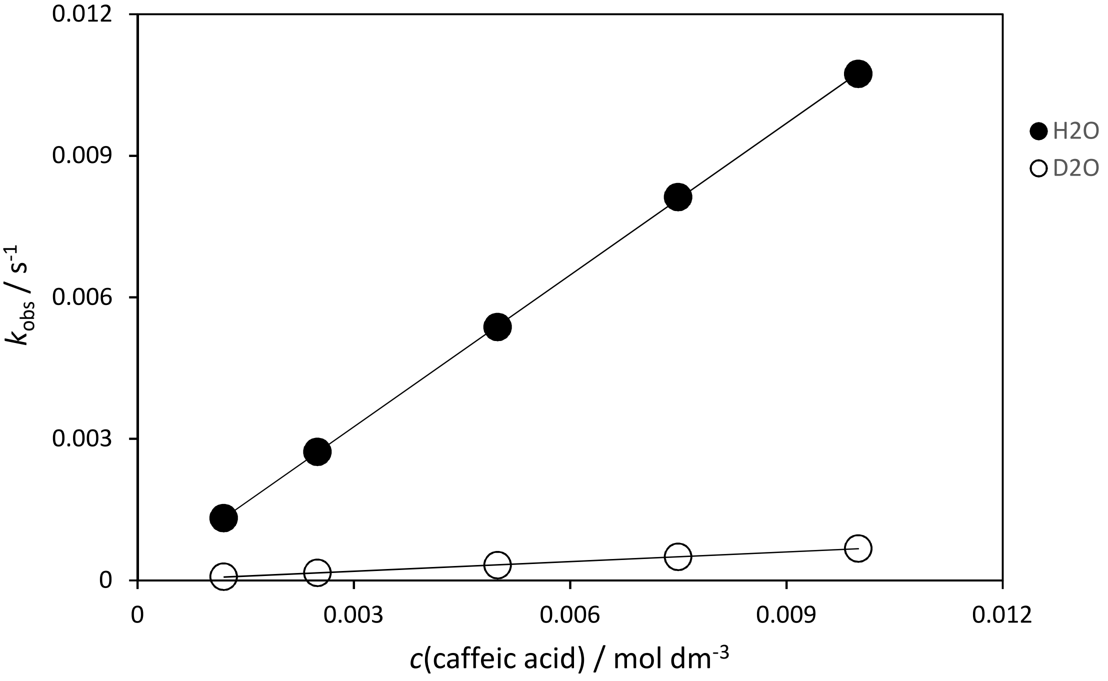

Figure S7. The plot of pseudo-first order constants (k_{obs}) *vs* concentration of caffeic acid for the reaction of caffeic acid and DPPH• radical in 1,4-dioxane-water solvent mixture (0.95 : 0.05 *v*/*v*) at 25 °C (H: R^2=0.9999; D: R^2=0.9995); *c*(DPPH•) = 0.00008 mol dm^{-3}; *c*(HCl or DCl) = 0.0005 mol dm^{-3}. Data from Table S7.

Thermodynamic Activation Parameters

Reaction of Hydroxytyrosol and DPPH• Radical

Table S8. Second Order Rate Constants for Reaction of Hydroxytyrosol and DPPH• Radical in 1,4-Dioxane-Water Solvent Mixture (0.99 : 0.01 *v*/*v*) at Various Temperatures.

t/°C	H_2O	D_2O		
	k_H / $M^{-1}s^{-1}$	k_D / $M^{-1}s^{-1}$	k_D* / $M^{-1}s^{-1}$	KIE
10.0	1.202 (0.039)	0.0698 (0.0039)	0.0630 (0.0017)	19.08 (0.81)
25.0	2.561 (0.073)	0.1755 (0.0328)	0.1602 (0.0094)	15.99 (1.04)
40.0	4.869 (0.151)	0.3812 (0.0159)	0.3543 (0.0074)	13.74 (0.51)

c(HOTyr) = 0.0013 - 0.0029 mol dm^{-3}; *c*(DPPH•) = 0.00006 mol dm^{-3}

*observed rate constants are corrected for the content of H in 1,4-dioxane-D_2O solvent mixture

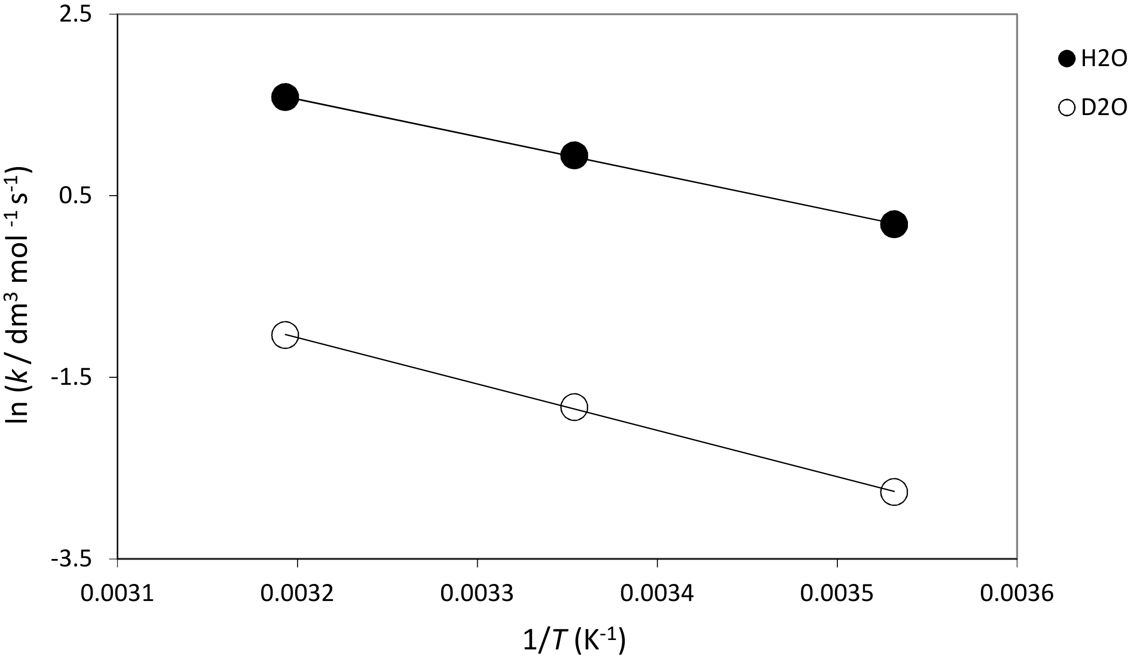

Figure S8. Arrhenius plot for the reaction of hydroxytyrosol and DPPH• radical in 1,4-dioxane-water solvent mixture (0.99 : 0.01 *v*/*v*) at various temperatures (H: R^2=0.9997; D: R^2=0.9997); *c*(DPPH•) = 0.00006 mol dm^{-3}. Data from Table S8.

Reaction of Homovanillyl Alcohol and DPPH• Radical

Table S9. Second Order Rate Constants for Reaction of Homovanillyl Alcohol and DPPH• Radical in 1,4-Dioxane-Water Solvent Mixture (0.99 : 0.01 *v*/*v*) at Various Temperatures.

	H_2O	D_2O		
t/°C	k_H / $M^{-1}s^{-1}$	$k_D \cdot 10^2$ / $M^{-1}s^{-1}$	$k_D^* \cdot 10^3$ / $M^{-1}s^{-1}$	KIE
10.0	0.0738 (0.0014)*	0.7265 (0.0028)*	4.491 (0.022)	15.42 (0.31)
17.5	0.1064 (0.0023)	1.145 (0.016)	7.205 (0.12)	14.76 (0.40)
25.0	0.1498 (0.0017)*	1.707 (0.031)*	10.83 (0.17)	14.29 (0.28)
32.5	0.2160 (0.0041)	2.460 (0.068)	16.05 (0.51)	13.46 (0.50)
40.0	0.2860 (0.0102)*	3.638 (0.025)*	24.78 (0.15)	11.94 (0.42)

c(HOTyr) = 0.025 mol dm^{-3}; *c*(DPPH•) = 0.00006 mol dm^{-3}.

* reaction rate constants are calculated from the slope of the plot of k_{obs} *vs* concentration

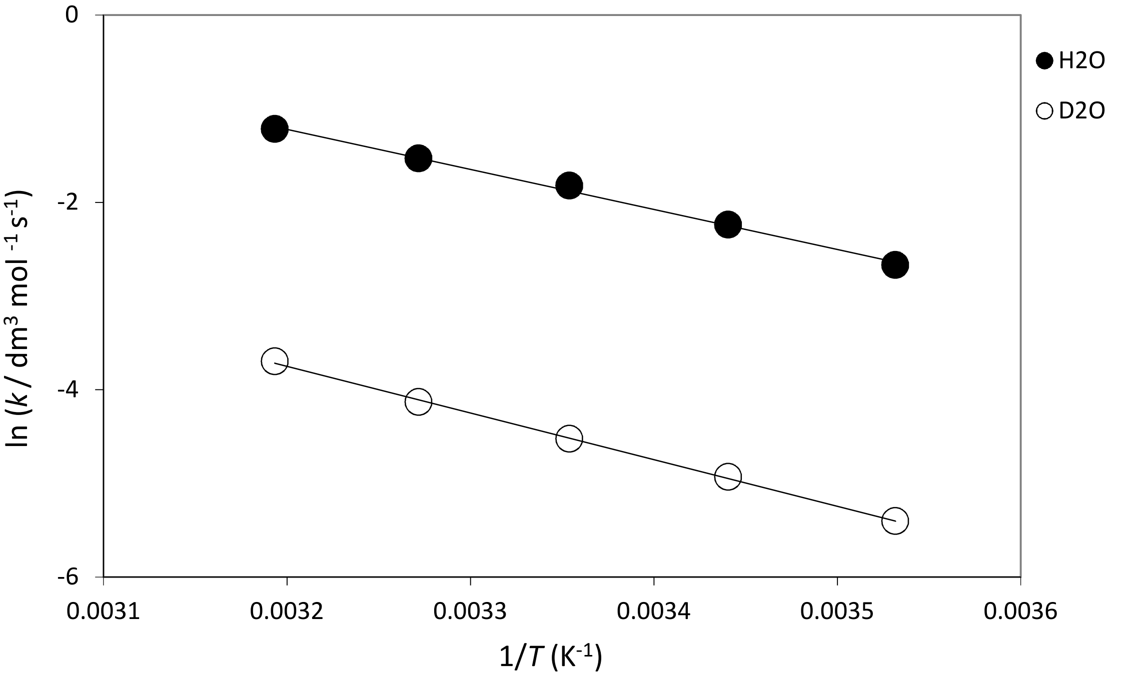

Figure S9. Arrhenius plot for the reaction of homovanillyl alcohol and DPPH• radical in 1,4-dioxane-water solvent mixture (0.99 : 0.01 *v*/*v*) at various temperatures (H: R^2=0.9963; D: R^2=0.9993); *c*(DPPH•) = 0.00006 mol dm^{-3}. Data from Table S9.

Reaction of Caffeic Acid and DPPH• Radical

Table S10. Second Order Rate Constants for Reaction of Caffeic Acid and DPPH• Radical in 1,4-Dioxane-Water Solvent Mixture (0.95 : 0.05 *v*/*v*) at Various Temperatures.

t/°C	H_2O k_H / $M^{-1}s^{-1}$	D_2O k_D / $M^{-1}s^{-1}$	KIE
14.0	0.262 (0.007)	0.0150 (0.0004)	17.5 (0.7)
20.0	0.370 (0.007)	0.0224 (0.0010)	16.5 (0.8)
25.0	0.537 (0.009)	0.0322 (0.0007)	16.7 (0.5)
32.0	0.782 (0.015)	0.0552 (0.0041)	14.2 (1.1)
39.0	1.111 (0.006)	0.0831 (0.0045)	13.4 (0.7)
45.0	1.534 (0.040)	0.1287 (0.0025)	11.9 (0.4)

c(DPPH•) = 0.00008 mol dm^{-3}; c(CA) = 0.005 mol dm^{-3}, c(HCl or DCl) = 0.0005 mol dm^{-3}

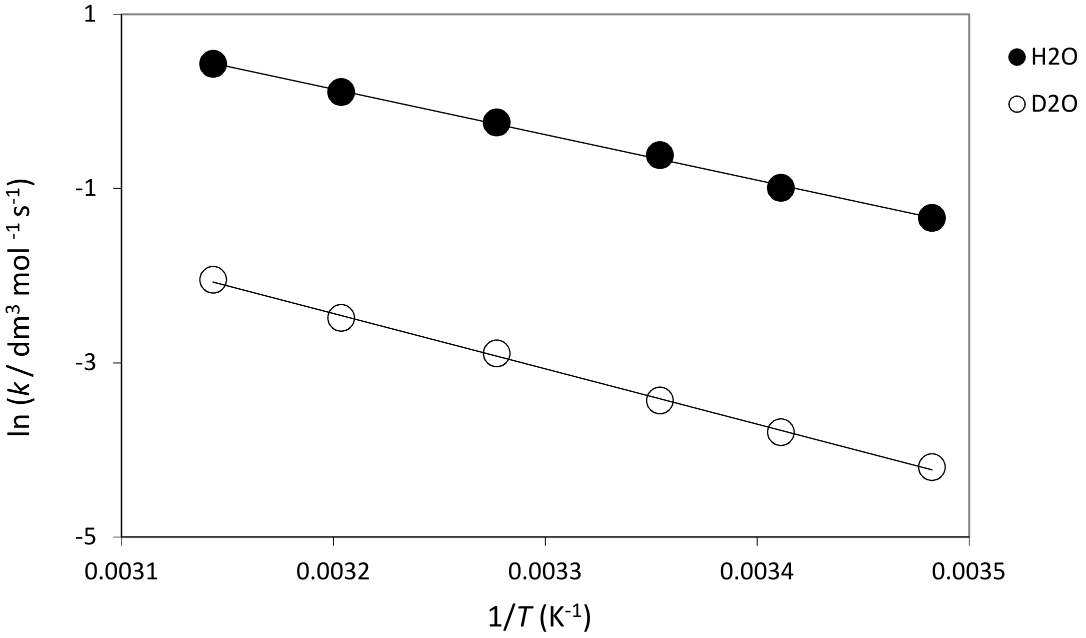

Figure S10. Arrhenius plot for the reaction of caffeic acid and DPPH• radical in 1,4-dioxane-water solvent mixture (0.95 : 0.05 *v*/*v*) at various temperatures (H: R^2=0.9984; D: R^2=0.9987); c(DPPH•) = 0.00008 mol dm^{-3}; c(CA) = 0.005 mol dm^{-3}, c(HCl or DCl) = 0.0005 mol dm^{-3}. Data from Table S10.

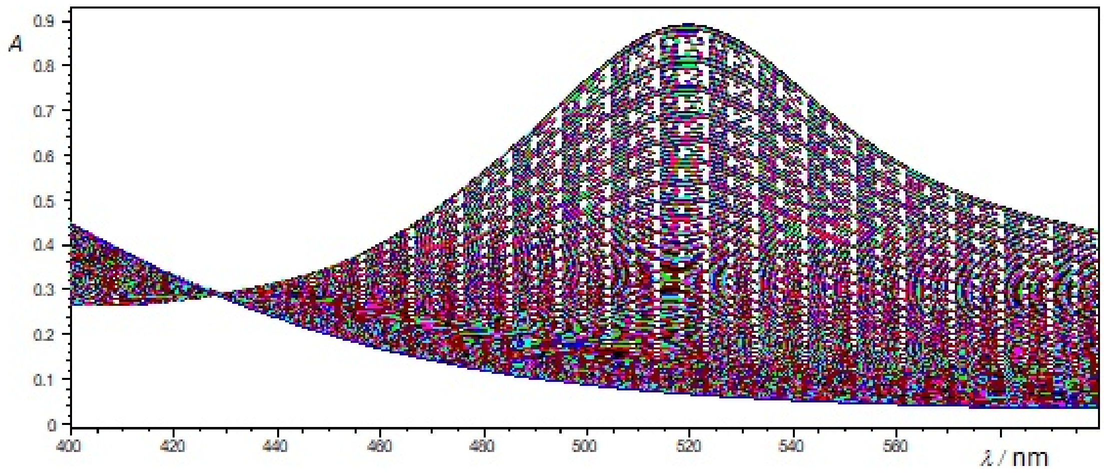

Figure S11. Change in UV/Vis spectra of the reaction mixture of 0.0012 mol dm^{-3} caffeic acid and 0.00008 mol dm^{-3} DPPH•, in presence of $c(HCl)$ = 0.0005 mol dm^{-3}, in 1,4-dioxane-water solvent mixture (0.95 : 0.05 *v*/*v*), at 25.0°C.

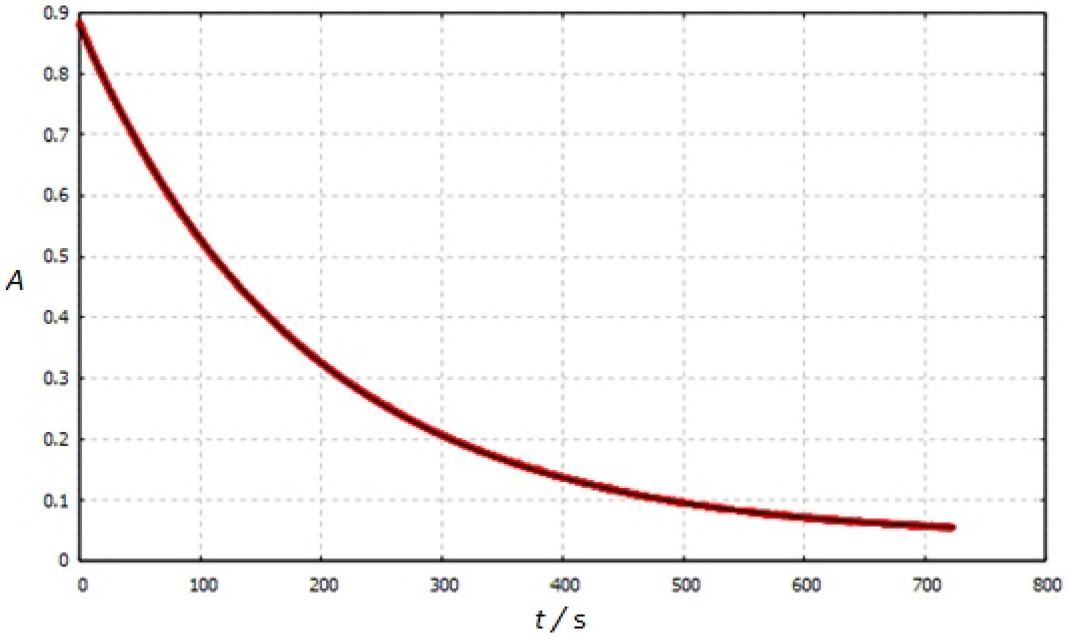

Figure S12: Kinetic trace at 520 nm for the reaction of 0.005 mol dm^{-3} caffeic acid and 0.00008 mol dm^{-3} DPPH• in presence of $c(HCl)$ = 0.0005 mol dm^{-3}, in 1,4-dioxane-water solvent mixture (0.95:0.05 *v*/*v*), at 25.0°C.

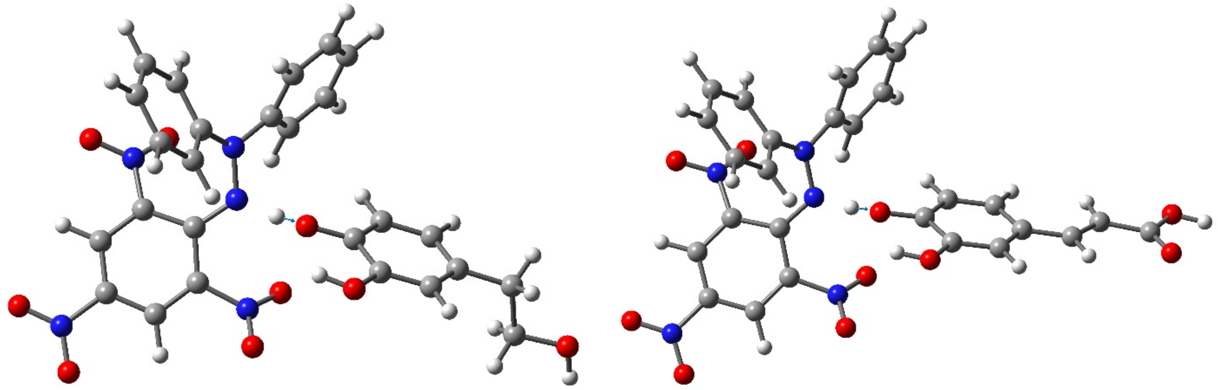

(a) (b)

Figure S13. Transition state structures for proposed hydrogen transfer to DPPH• radical from (a) hydroxytyrosol and (b) caffeic acid obtained from the DFT calculations. Normal mode displacement vectors shown (blue arrow) for unique imaginary frequencies of 1646.6*i* and 1689.0*i* cm^{-1} scaled by factor of 0.9679 [68] for hydroxytyrosol and caffeic acid respectively, are associated with a motion of the H atom of the O-H moiety of phenols to the N atom of DPPH• radical.

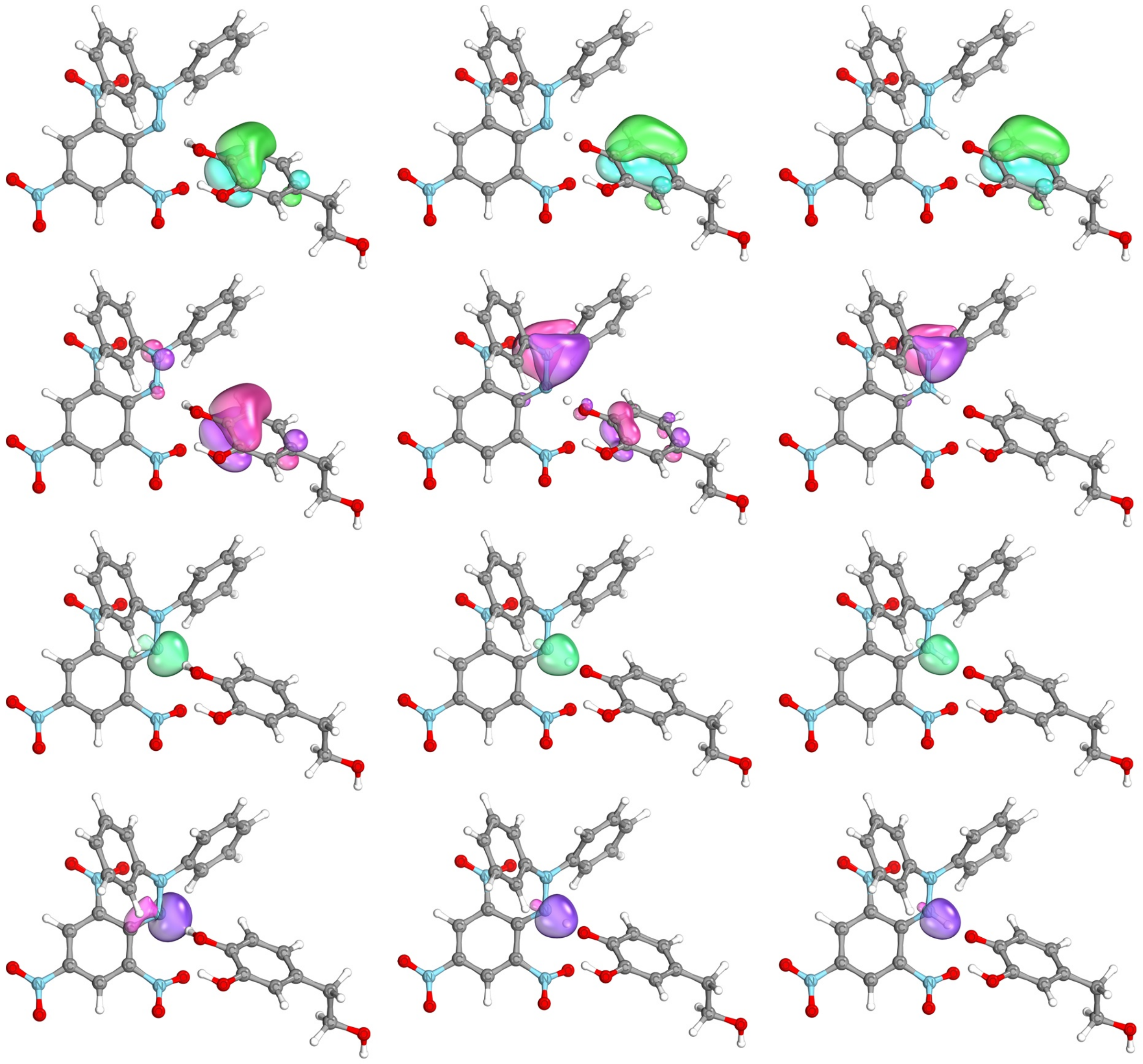

A **TS** **B**

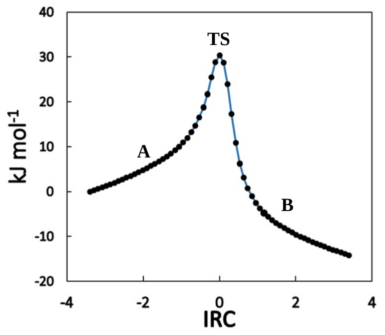

Figure S14. Changes in energy plotted along the IRC path (left), α (green) and β (purple) intrinsic bond orbitals (IBO) involved in hydrogen transfer from hydroxytyrosol to DPPH• radical plotted along the intrinsic reaction coordinate (IRC) obtained from the DFT calculations. For IBO analysis along the entire IRC see also the corresponding video files.

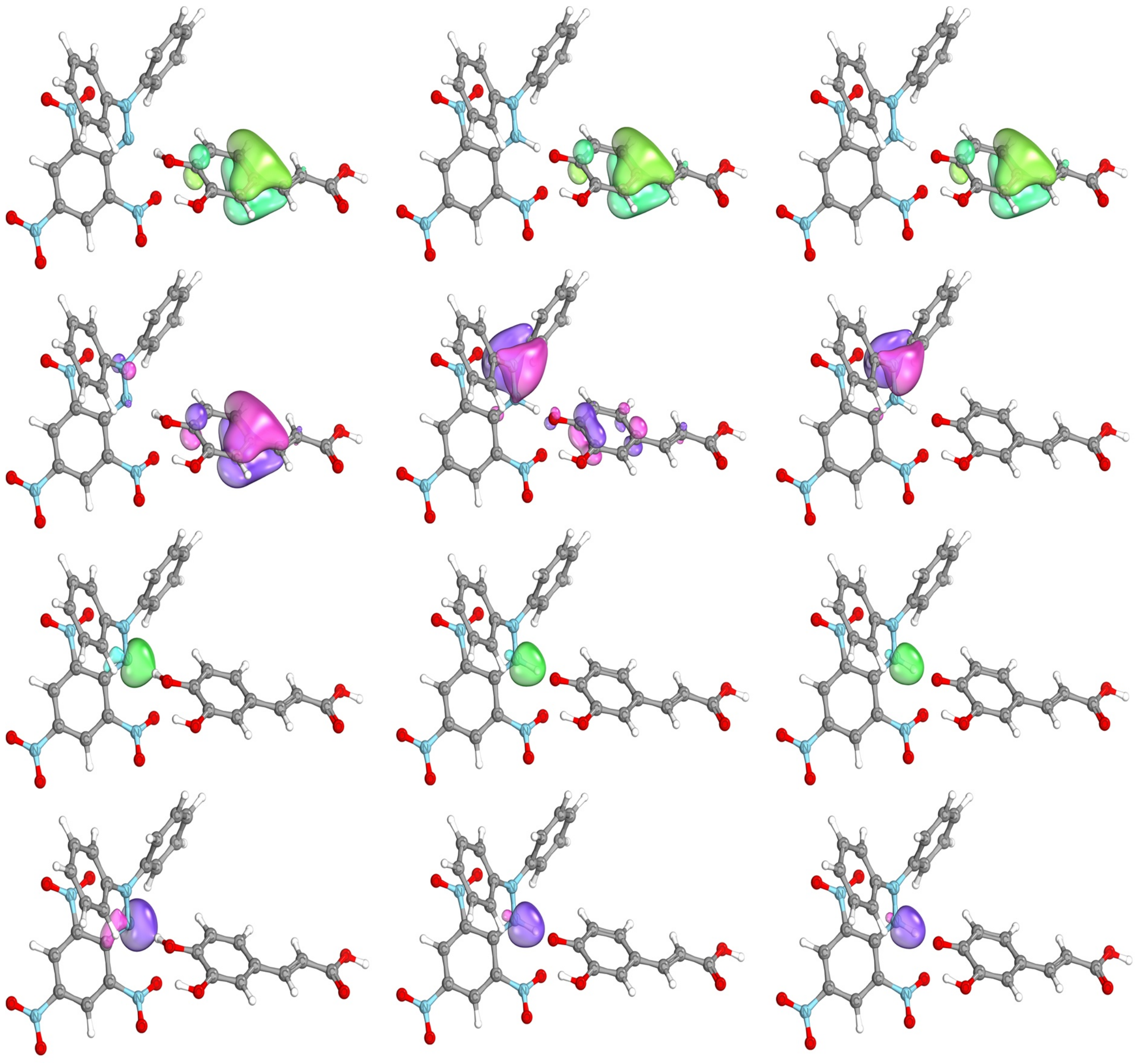

A **TS** **B**

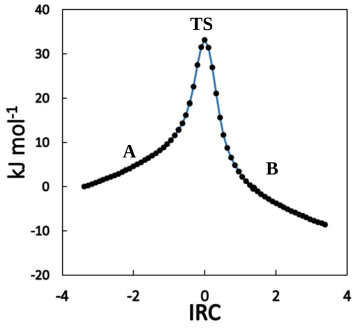

Figure S15. Changes in energy plotted along the IRC path (left), α (green) and β (purple) intrinsic bond orbitals (IBO) involved in hydrogen transfer from caffeic acid to DPPH• radical plotted along the intrinsic reaction coordinate (IRC) obtained from the DFT calculations. For IBO analysis along the entire IRC see also the corresponding video files.

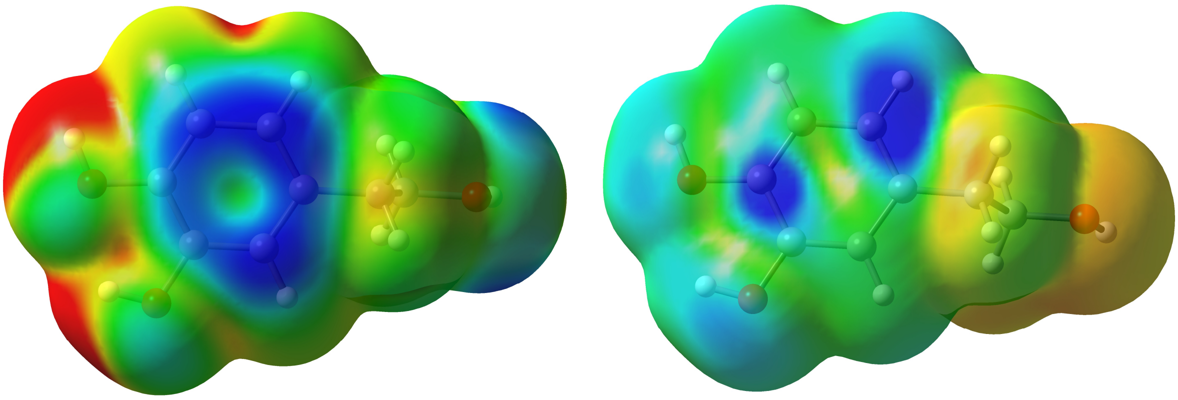

(a) (b)

Figure S16. The average local ionization energy $\bar{I}(r)$ (a) and the electron donor Fukui function $f^{-}(r)$ (b) plotted on 0.001 a.u. electron density isosurface of hydroxytyrosol. The color scales corresponds to the range of values 13.6 eV (*red*) to 4.5 eV (*blue*) for $\bar{I}(r)$ and 0.0 a.u. (*red*) to 0.0003 a.u. (*blue*) for $f^{-}(r)$. The lowest values of $\bar{I}(r)$ (blue areas) and highest values of $f^{-}(r)$ (blue areas) indicate the most preferred sites for donating electron. The lowest average local ionization energy $\bar{I}_{S,min}$ have value of 8.69 eV.

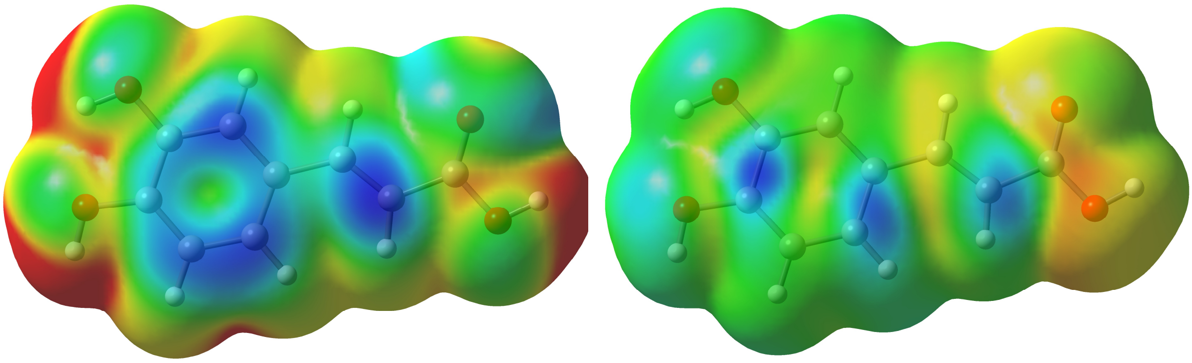

(a) (b)

Figure S17. The average local ionization energy $\bar{I}(r)$ (a) and the electron donor Fukui function $f^{-}(r)$ (b) plotted on 0.001 a.u. electron density isosurface of caffeic acid. The color scales corresponds to the range of values 13.6 eV (*red*) to 4.5 eV (*blue*) for $\bar{I}(r)$ and 0.0 a.u. (*red*) to 0.0003 a.u. (*blue*) for $f^{-}(r)$. The lowest values of $\bar{I}(r)$ (blue areas) and highest values of $f^{-}(r)$ (blue areas) indicate the most preferred sites for donating electron. The lowest average local ionization energy $\bar{I}_{S,min}$ have value of 8.97 eV.

Table S11. The reactant, transition state and product total energies and coordinates of the optimized structures at (U)B3LYP/6-311++G(2d,2p) level by using solvent polarizable continuum model (PCM) of self-consistent reaction field (SCRF) method with dioxane as a solvent.

DPPH•				H-DPPH [a]			
E_{tot} = -1418.313928 Hartree				E_{tot} = -1418.947198 Hartree			
C	-0.615276	2.403035	2.602633	C	-1.619864	3.532611	1.268904
C	-0.754411	1.175850	1.967433	C	-1.537044	2.144679	1.261045
C	-1.423263	1.112811	0.744358	C	-1.590254	1.454637	0.050971
C	-1.957964	2.260886	0.162118	C	-1.734177	2.151165	-1.145225
C	-1.812936	3.481183	0.809130	C	-1.832423	3.538858	-1.129348
C	-1.141722	3.556014	2.026508	C	-1.771111	4.232232	0.075128
H	-0.104749	2.454750	3.553832	H	-1.574419	4.064619	2.208915
H	-0.369451	0.271913	2.417012	H	-1.433253	1.609676	2.194743
H	-2.461722	2.198855	-0.790914	H	-1.764874	1.608135	-2.078136
H	-2.215193	4.374986	0.354088	H	-1.944140	4.075774	-2.060907
H	-1.029722	4.508855	2.524182	H	-1.837407	5.311122	0.084427
N	-1.572302	-0.162796	0.094337	N	-1.493244	0.008146	0.003481
N	-0.570022	-1.027794	0.011125	N	-0.470080	-0.512783	0.800169
C	-2.853374	-0.654153	-0.264696	C	-2.687329	-0.777134	0.008906
C	-4.004389	-0.187397	0.379091	C	-3.933500	-0.183710	-0.215936
C	-2.949744	-1.633994	-1.257852	C	-2.616786	-2.159290	0.211380
C	-5.242732	-0.701007	0.021289	C	-5.081340	-0.966749	-0.246420
H	-3.931474	0.551861	1.161396	H	-4.012734	0.881273	-0.365232
C	-4.193945	-2.139107	-1.601785	C	-3.773652	-2.927677	0.186146
H	-2.055034	-1.976132	-1.752922	H	-1.662128	-2.632939	0.375569
C	-5.344686	-1.675671	-0.967688	C	-5.013057	-2.340827	-0.045058
H	-6.129423	-0.344935	0.526415	H	-6.036053	-0.490358	-0.422172
H	-4.265985	-2.890971	-2.374916	H	-3.698504	-3.994842	0.343425
H	-6.312440	-2.071078	-1.241879	H	-5.909803	-2.943491	-0.066451
C	0.712285	-0.623457	-0.138744	C	0.813708	-0.510635	0.385544
C	1.708615	-1.437848	0.475416	C	1.919315	-0.480190	1.294908
C	1.243240	0.449070	-0.922596	C	1.177878	-0.488869	-0.990120
C	3.058920	-1.227477	0.361471	C	3.229750	-0.352570	0.857280
C	2.599415	0.708527	-1.005351	C	2.467730	-0.335905	-1.426900
C	3.498978	-0.124005	-0.364156	C	3.496304	-0.260506	-0.490185
H	3.764030	-1.883106	0.846548	H	4.027165	-0.310693	1.579984
H	2.949877	1.532829	-1.604412	H	2.687672	-0.311553	-2.482506
N	0.402343	1.244021	-1.823116	N	0.185887	-0.781871	-2.046709
O	-0.589609	0.703312	-2.302565	O	-0.398428	-1.851027	-1.963947
O	0.766105	2.385425	-2.083769	O	0.090995	0.012970	-2.970640
N	4.928298	0.142676	-0.469794	N	4.874933	-0.109904	-0.947148
O	5.694807	-0.634246	0.093361	O	5.759416	-0.082448	-0.098427
O	5.281730	1.127138	-1.112278	O	5.063121	-0.018299	-2.156351
N	1.291157	-2.544245	1.357210	N	1.731176	-0.530899	2.738633
O	0.483777	-2.293751	2.240686	O	0.600960	-0.789495	3.176959
O	1.818551	-3.631681	1.168785	O	2.695396	-0.321475	3.460326
				H	-0.618780	-0.598086	1.799868

[a] Product formed from DPPH• after accepting hydrogen from the phenols.

Table S11. Continued …

Hydroxytyrosol				Hydroxytyrosol radical [a]			
E_{tot} = -536.721621 Hartree				E_{tot} = -536.092332 Hartree			
C	-0.647204	-0.225168	0.423001	C	0.592806	0.111192	0.429422
C	0.156870	0.914606	0.334031	C	-0.249279	-0.993493	0.313301
C	-0.054261	-1.473487	0.237959	C	0.060870	1.424376	0.269627
C	1.513990	0.814071	0.067280	C	-1.592397	-0.805671	0.040636
C	1.308896	-1.584367	-0.030073	C	-1.260745	1.640174	0.000933
H	-0.653676	-2.371032	0.309569	H	0.731489	2.267220	0.374314
C	2.092877	-0.445553	-0.116615	C	-2.163654	0.529669	-0.130977
H	1.762199	-2.558518	-0.167151	H	-1.665089	2.635721	-0.114493
O	3.446384	-0.447309	-0.371765	O	-3.390935	0.618134	-0.375156
H	3.767852	-1.348982	-0.463293	H	0.133133	-1.997276	0.439183
H	-0.263459	1.900841	0.481042	O	-2.441881	-1.827567	-0.078246
O	2.274184	1.947312	-0.007569	H	-3.311455	-1.420266	-0.265194
H	3.187511	1.691842	-0.185612	C	2.063260	-0.071807	0.688602
C	-2.130484	-0.096950	0.681273	H	2.435591	0.733985	1.323327
H	-2.507506	-1.001339	1.160647	H	2.242266	-1.011342	1.211444
H	-2.326889	0.732403	1.362703	C	2.881099	-0.075979	-0.604873
C	-2.924109	0.136283	-0.600743	H	2.725504	0.859206	-1.150740
H	-2.767585	-0.697218	-1.292277	H	2.557500	-0.901442	-1.245498
H	-2.580104	1.051747	-1.091280	O	4.254421	-0.225402	-0.242752
O	-4.308173	0.244738	-0.250801	H	4.786455	-0.237896	-1.043145
H	-4.816953	0.405712	-1.049955				

[a] Product formed from hydroxytyrosol in reaction of hydrogen transfer to DPPH•.

Homovanillyl alcohol				Homovanillyl alcohol radical [a]			
E_{tot} = -576.026552 Hartree				E_{tot} = -575.391802 Hartree			
C	-0.859352	-0.406229	0.425972	C	-0.818500	-0.308321	0.426071
C	0.089309	0.617445	0.300340	C	0.183025	0.658897	0.289790
C	-0.436540	-1.721266	0.276315	C	-0.487585	-1.679533	0.286384
C	1.426160	0.343224	0.031426	C	1.494553	0.298168	0.017348
C	0.900836	-2.009843	0.005310	C	0.792336	-2.073085	0.017918
H	-1.144390	-2.533260	0.376038	H	-1.268363	-2.419898	0.403082
C	1.835247	-0.994841	-0.119618	C	1.866000	-1.118283	-0.135932
H	1.221839	-3.038707	-0.105155	H	1.056590	-3.116125	-0.085721
O	3.160188	-1.222801	-0.380041	O	3.039057	-1.462849	-0.379024
H	3.309373	-2.170956	-0.448073	H	-0.082242	1.699135	0.406949
H	-0.229486	1.641646	0.425035	O	2.512104	1.152588	-0.124648
O	2.405261	1.281403	-0.097239	C	-2.242678	0.102610	0.685870
C	-2.310603	-0.073045	0.683694	H	-2.725991	-0.617375	1.348389
H	-2.796407	-0.894034	1.212835	H	-2.274214	1.073888	1.180268
H	-2.391111	0.809331	1.321074	C	-3.064019	0.190541	-0.601771
C	-3.082661	0.193557	-0.605043	H	-3.069842	-0.778281	-1.109390
H	-3.052329	-0.692262	-1.246456	H	-2.617732	0.925526	-1.278160
H	-2.622427	1.022292	-1.151710	O	-4.390718	0.578202	-0.240233
O	-4.433709	0.513929	-0.254991	H	-4.929480	0.618079	-1.035199
H	-4.934298	0.664547	-1.061298	C	2.267866	2.554031	0.012773
C	2.046942	2.650019	0.049343	H	1.559789	2.900880	-0.740604
H	1.317514	2.953005	-0.704743	H	1.893156	2.786868	1.010039
H	1.646684	2.851792	1.045061	H	3.228803	3.033617	-0.139129
H	2.965095	3.212236	-0.090869				

[a] Product formed from Homovanillyl alcohol in reaction of hydrogen transfer to DPPH•.

Caffeic acid				Caffeic acid radical [a]			
E_{tot} = -648.896476 Hartree				E_{tot} = -648.267115 Hartree			
C	0.088388	0.031857	-0.000045	C	-0.138977	0.060259	0.000008
C	1.039701	1.064176	0.000004	C	-1.094157	1.090124	-0.000008
C	0.541704	-1.295489	-0.000030	C	-0.576958	-1.307918	-0.000016
C	2.396120	0.788973	0.000082	C	-2.436313	0.783421	-0.000058
C	1.898752	-1.576317	0.000042	C	-1.894852	-1.635769	-0.000061
H	-0.161315	-2.114866	-0.000086	H	0.162253	-2.095149	0.000014
C	2.828119	-0.542080	0.000102	C	-2.907574	-0.606318	-0.000089
H	2.244030	-2.602445	0.000048	H	-2.227196	-2.663906	-0.000074
O	4.184968	-0.724987	0.000176	O	-4.136746	-0.810628	-0.000133
H	4.402144	-1.662420	0.000180	H	-0.781291	2.125071	0.000014
H	0.723754	2.098342	-0.000012	O	-3.381959	1.723308	-0.000080
O	3.293984	1.815053	0.000135	C	1.256580	0.421890	0.000048
C	-1.322235	0.394195	-0.000109	H	1.475143	1.483264	0.000126
H	-1.535280	1.457299	-0.000234	C	2.315970	-0.413262	-0.000017
C	-2.383306	-0.428333	-0.000020	H	2.220599	-1.488310	-0.000112
H	-2.293421	-1.504353	0.000126	H	-4.232709	1.241872	-0.000115
H	4.186431	1.448002	0.000180	C	3.685214	0.125687	0.000022
C	-3.745233	0.115410	-0.000102	O	3.992013	1.297626	0.000248
O	-4.060503	1.287020	-0.000292	O	4.607709	-0.867906	0.000085
O	-4.677627	-0.875545	0.000020	H	5.481464	-0.450717	0.000218
H	-5.544978	-0.446236	-0.000058				

[a] Product formed from Caffeic acid in reaction of hydrogen transfer to DPPH•.

Table S11. Continued … coordinates of transition structure (TS) for hydrogen transfer from Hydroxytyrosol to DPPH• radical:

E_{tot} = -1955.021395 Hartree			
C	-3.882874	-1.509168	-0.778196
C	-2.969116	-0.477301	-0.867889
C	-1.569595	-0.653435	-0.572840
C	-1.190745	-2.039844	-0.461186
C	-2.095028	-3.067215	-0.374679
C	-3.454146	-2.793170	-0.493181
H	-4.924320	-1.309911	-0.969858
H	-1.749747	-4.078478	-0.235158
N	-0.649256	0.286541	-0.311080
N	-1.008226	1.588148	-0.030305
C	-0.213900	2.585707	-0.628452
C	0.282452	2.386183	-1.924531
C	0.105353	3.764844	0.061101
C	1.072217	3.358231	-2.518962
H	0.025014	1.484743	-2.457218
C	0.892076	4.729336	-0.549228
H	-0.244921	3.913134	1.070766
C	1.378786	4.535684	-1.840311
H	1.438850	3.200328	-3.523679
H	1.138508	5.631231	-0.006549
H	1.991120	5.292082	-2.310208
C	-1.930511	1.862024	1.019843
C	-2.807946	2.945208	0.906989
C	-1.972475	1.046827	2.155253
C	-3.707634	3.212827	1.928646
H	-2.797012	3.553806	0.015252
C	-2.884355	1.318769	3.165308
H	-1.282303	0.222527	2.252895
C	-3.752776	2.402101	3.059563
H	-4.388809	4.046131	1.829885
H	-2.907131	0.688586	4.043292
H	-4.460919	2.609587	3.849079
N	-3.495646	0.765789	-1.426465
O	-4.683975	1.022124	-1.257344
O	-2.726986	1.455527	-2.092005
N	-4.418683	-3.873499	-0.397102
O	-3.989611	-5.007598	-0.191556
O	-5.609510	-3.594907	-0.520697
N	0.222083	-2.435520	-0.463460
O	0.974019	-1.843580	-1.229299
O	0.555768	-3.367241	0.260360
C	5.344743	-0.287762	0.462624
C	4.764386	-0.873499	1.583324
C	4.518117	0.400142	-0.456626
C	3.394975	-0.787153	1.784977
C	3.158381	0.505699	-0.266794
H	4.969682	0.855440	-1.327830
C	2.558143	-0.082162	0.862214
H	2.538081	1.036321	-0.973238
O	1.282807	-0.011270	1.170959
H	0.464154	0.070774	0.328842
H	5.365071	-1.405511	2.308199
O	2.827794	-1.350835	2.865929
H	1.872409	-1.190973	2.802830
C	6.822001	-0.419642	0.203496
H	7.366323	-0.504383	1.144449
H	7.194260	0.467542	-0.310972
C	7.149194	-1.642844	-0.654420
H	6.621303	-1.581670	-1.610570
H	6.821327	-2.552706	-0.143689
O	8.563326	-1.659862	-0.859564
H	8.789753	-2.429997	-1.388141

Table S11. Continued … coordinates of transition structure (TS) for hydrogen transfer from homovanillyl alcohol to DPPH• radical:

E_{tot} = -1994.325216 Hartree			
C	-3.932736	-1.532079	-0.838747
C	-3.070797	-0.456936	-0.914857
C	-1.648543	-0.586217	-0.696270
C	-1.202273	-1.961372	-0.718011
C	-2.059229	-3.029643	-0.646977
C	-3.433937	-2.810966	-0.657802
H	-4.990552	-1.369764	-0.963678
H	-1.664446	-4.031218	-0.605951
N	-0.769170	0.371959	-0.390318
N	-1.183533	1.629814	0.003321
C	-0.456793	2.709596	-0.532549
C	0.031168	2.629133	-1.844665
C	-0.195344	3.857279	0.230398
C	0.756436	3.683083	-2.379853
H	-0.181989	1.752520	-2.435384
C	0.525559	4.905378	-0.321733
H	-0.540678	3.918902	1.250698
C	1.005354	4.828553	-1.627294
H	1.116764	3.613793	-3.396965
H	0.725065	5.782325	0.278382
H	1.566031	5.649425	-2.051316
C	-2.067625	1.766746	1.113655
C	-3.031067	2.779481	1.110626
C	-1.982858	0.888360	2.198017
C	-3.893270	2.915034	2.189692
H	-3.113224	3.438225	0.258891
C	-2.859678	1.026337	3.265455
H	-1.222695	0.121582	2.213120
C	-3.815091	2.039185	3.268950
H	-4.641311	3.695176	2.175833
H	-2.785567	0.347021	4.103157
H	-4.494581	2.142483	4.103059
N	-3.671373	0.788027	-1.381250
O	-4.868075	0.973265	-1.173622
O	-2.954350	1.558428	-2.016143
N	-4.342912	-3.936074	-0.573915
O	-3.856340	-5.061606	-0.466249
O	-5.550995	-3.706486	-0.608856
N	0.217835	-2.293901	-0.853021
O	0.911330	-1.557731	-1.548193
O	0.622988	-3.317294	-0.311241
C	5.232105	-0.072181	0.194157
C	4.717335	-0.778230	1.280596
C	4.348553	0.662232	-0.626642
C	3.353764	-0.771504	1.557138
C	2.998987	0.691588	-0.372544
H	4.745654	1.211993	-1.469371
C	2.452143	-0.014458	0.723187
H	2.327353	1.254576	-1.003010
O	1.190255	0.007513	1.035488
H	0.376705	0.156132	0.212793
H	5.392792	-1.338275	1.909155
O	2.772309	-1.419523	2.574992
C	6.701544	-0.125194	-0.126766
H	7.280695	-0.326135	0.774886
H	7.034520	0.836065	-0.521425
C	7.028271	-1.204980	-1.160130
H	6.476155	-1.017369	-2.085372
H	6.728749	-2.185916	-0.779866
O	8.436748	-1.162752	-1.395244
H	8.660629	-1.826738	-2.053182
C	3.583642	-2.208689	3.448693
H	4.084120	-3.005101	2.897609
H	4.320050	-1.588174	3.960221
H	2.899161	-2.638025	4.172194

Table S11. Continued … coordinates of transition structure (TS) for hydrogen transfer from caffeic acid to DPPH• radical:

E_{tot} = -2067.196106 Hartree			
C	-4.399099	-1.306005	-0.903899
C	-3.411142	-0.340194	-0.931784
C	-2.042565	-0.621980	-0.591724
C	-1.768648	-2.029577	-0.480557
C	-2.745181	-2.992988	-0.454635
C	-4.073523	-2.621582	-0.631040
H	-5.415341	-1.029388	-1.131450
H	-2.478177	-4.027690	-0.314166
N	-1.062150	0.252502	-0.298177
N	-1.337580	1.566690	0.008304
C	-0.466494	2.521969	-0.547499
C	0.046353	2.318898	-1.837289
C	-0.088577	3.661554	0.179257
C	0.910258	3.250891	-2.389735
H	-0.258649	1.449809	-2.397822
C	0.773609	4.585216	-0.389212
H	-0.450566	3.806891	1.185144
C	1.276160	4.389306	-1.674323
H	1.289090	3.094076	-3.389939
H	1.067041	5.455535	0.180671
H	1.947404	5.114546	-2.111640
C	-2.269232	1.879616	1.038944
C	-3.069333	3.021284	0.926160
C	-2.393561	1.045118	2.154444
C	-3.976826	3.326679	1.929859
H	-2.994522	3.644931	0.048032
C	-3.311326	1.357450	3.146687
H	-1.759437	0.177007	2.252397
C	-4.104109	2.497792	3.041234
H	-4.599514	4.204540	1.832352
H	-3.397915	0.714387	4.011108
H	-4.817679	2.735809	3.817075
N	-3.830661	0.952213	-1.474100
O	-4.999930	1.290995	-1.325721
O	-2.997051	1.596584	-2.105306
N	-5.116911	-3.633980	-0.599731
O	-4.777428	-4.797358	-0.396827
O	-6.276753	-3.268120	-0.772401
N	-0.387209	-2.525054	-0.416256
O	0.434320	-1.997598	-1.156493
O	-0.153880	-3.466591	0.331525
C	4.878369	-0.669688	0.600350
C	4.236017	-1.189219	1.733557
C	4.103099	0.021036	-0.366226
C	2.874725	-1.030327	1.901999
C	2.750645	0.192312	-0.204430
H	4.578865	0.418837	-1.250134
C	2.096211	-0.320236	0.935969
H	2.168075	0.718028	-0.945618
O	0.823040	-0.173278	1.205142
H	0.006034	-0.041834	0.333347
H	4.798610	-1.725851	2.484654
O	2.254528	-1.526567	2.991112
C	6.306550	-0.867635	0.466356
H	6.787640	-1.414501	1.269242
C	7.099530	-0.449878	-0.537870
H	6.729277	0.100905	-1.389238
H	1.310830	-1.322196	2.897501
C	8.539932	-0.734650	-0.516163
O	9.144041	-1.334214	0.347322
O	9.161500	-0.233299	-1.615193
H	10.100351	-0.455841	-1.537659
